# Effects of Fly Ash–Metakaolin Composition and Sodium Silicate-to-Sodium Hydroxide (SS/SH) Ratio on Hybrid Geopolymer Concrete After Elevated-Temperature Exposure: Mechanical, Microstructural, and Machine-Learning Assessment

**DOI:** 10.3390/ma19214554

**Published:** 2026-10-28

**Authors:** Yasin Onuralp Özkılıç, Fatih Yılmaz, Sergey A. Stel’makh, Ali İhsan Çelik, Sadik Alper Yildizel

**Affiliations:** 1 Department of Civil Engineering, Necmettin Erbakan University, Konya 42090, Türkiye; fatihyilmazfthylmz@gmail.com; 2 Department of Technical Sciences, Western Caspian University, Baku 1001, Azerbaijan; 3 Department of Civil Engineering, Faculty of Engineering and Natural Sciences, Dogus University, Dudullu-Ümraniye, Istanbul 34775, Türkiye; 4 Department of Unique Buildings and Constructions Engineering, Don State Technical University, Gagarin Sq. 1, 344003 Rostov-on-Don, Russia; sstelmah@donstu.ru; 5 Department of Construction, Tomarza Mustafa Akincioglu Vocational School, Kayseri University, Kayseri 38940, Türkiye; acelik@kayseri.edu.tr; 6 Department of Civil Engineering, Engineering Faculty, Karamanoglu Mehmetbey University, Karaman 70200, Türkiye

**Keywords:** geopolymer concrete, metakaolin, alkali activator composition, elevated-temperature exposure, residual mechanical properties, microstructural characterization, machine learning, Gaussian process regression, uncertainty quantification, multi-objective optimization

## Abstract

This study investigates the coupled effects of metakaolin (MK) replacement and the sodium silicate-to-sodium hydroxide (SS/SH) ratio on the fresh-state, mechanical, thermal, and microstructural properties of ambient-cured hybrid geopolymer concrete. Fly ash was replaced by MK at levels of 0%, 25%, 50%, 75%, and 100%, and SS/SH ratios of 2.0, 2.5, and 3.0 were adopted, producing 15 mixtures activated with a 12 M NaOH solution. Fresh-state slump was measured, and compressive and splitting tensile strengths were evaluated at ambient temperature and after exposure to 300 °C. XRD, FTIR, TGA/DTG, FE-SEM, and EDX analyses were also conducted to characterize the reaction matrix of a representative mixture (FA50–MK50–SS/SH2.5) after exposure to 300 °C. Increasing both MK content and SS/SH ratio reduced workability but substantially enhanced mechanical performance. At an SS/SH ratio of 3.0, replacing fly ash entirely with MK increased the ambient compressive strength from 20.17 to 39.59 MPa and the splitting tensile strength from 1.79 to 3.41 MPa, corresponding to improvements of 96.27% and 90.25%, respectively. Following exposure to 300 °C, the corresponding residual strengths reached 13.81 MPa and 1.60 MPa, representing increases of 79.31% and 77.76%, respectively, over the MK-free mixture. Nevertheless, heating caused considerable absolute losses in strength across all mixtures. At an SS/SH ratio of 2.0, increasing MK content reduced thermal compressive strength loss from approximately 60% to 44.77%, indicating greater retention of compressive strength after exposure to 300 °C. Microstructural observations revealed a predominantly amorphous reaction matrix containing residual precursor particles, local voids, and microcracks. The representative FE-SEM images also showed locally continuous matrix regions and apparently well-bonded aggregate–binder interfaces. These observations are consistent with the development of alkali-activated reaction products and locally homogeneous matrix regions. Experimental evaluation on three newly tested and two independently reproduced compositions showed close agreement in the limited evaluation set (descriptive R^2^: 0.906–0.999; MAPE: 0.47–1.40%). The original 95% prediction intervals had a strict coverage of 90%, whereas a supplementary resolution-adjusted assessment gave a coverage of 95%. These results support the local predictive usefulness of the framework within the investigated design domain, and uncertainty-aware Pareto optimization identified MK content as the dominant design variable. Overall, MK content and activator composition jointly govern the balance among workability, strength development, and residual mechanical performance of hybrid geopolymer concrete after exposure to 300 °C.

## 1. Introduction

Ambient-cured fly ash–slag geopolymer concrete requires a balance between workability, strength development, and residual performance after heating. Cement-related emissions motivate investigation of alternative binders [1,2,3]. The foundational literature describes the chemistry and processing of geopolymers and alkali-activated materials [4,5,6,7], while studies and reviews address fly ash concrete, industrial by-product utilization, and sustainable construction [8,9,10,11,12,13,14]. Mixture modifications involving basalt powder, silica fume, metallic waste, and alkaline activation illustrate the formulation-specific nature of mechanical and fresh-state performance [15,16,17]. Recent precursor-comparison and curing studies further show that precursor chemistry and activation conditions must be considered together [18,19,20]. In the present FA–MK–GGBFS system, replacing fly ash with metakaolin (MK) changes the reactive aluminosilicate fraction while the slag dosage remains constant; the resulting strength benefit must therefore be assessed alongside workability and post-heating performance.

MK is a reactive aluminosilicate that can modify setting and matrix development [21,22,23,24,25,26]. Curing temperature and duration affect the strength and structure of MK-based geopolymers [27,28], while Si/Al ratio and formulation influence mechanical performance [29,30]. Activator composition is similarly important [31]. Reported favorable sodium silicate-to-sodium hydroxide (SS/SH) ratios differ among precursor systems: Morsy et al. [32] found a compressive strength maximum at 1.0 for a heat-cured fly ash binder, whereas Degirmenci [33] investigated formulation-dependent durability under chemical exposure. Studies of palm-oil fuel ash, fly ash–slag, calcined kaolin–cement, and waste-derived binders likewise show that SS/SH effects depend on precursor chemistry, NaOH concentration, and mixture proportions [34,35,36,37,38]. These findings do not establish a transferable optimum for ambient-cured FA–MK–slag concrete after heating.

The specific research problem is to determine how MK replacement and SS/SH jointly affect workability, the ambient compressive and splitting tensile strengths, and the residual values of those strengths after heating within a single controlled binder system. Existing studies either differ in precursor composition and curing conditions or emphasize individual variables and ambient compressive strength, limiting direct assessment of the coupled effects. Absolute residual strength and proportional strength retention must also be distinguished: a mixture with a high initial strength may retain a smaller fraction of its initial strength after heating, yet still exhibit a higher residual capacity. Evaluating both measures is therefore necessary to identify composition-dependent trade-offs.

This study tests the hypothesis that increasing MK replacement and silicate availability improves ambient and absolute residual strengths while reducing workability, with relative strength retention depending on their combined effects. A 5 × 3 factorial design comprises five MK replacement levels (0–100%) and three SS/SH ratios (2.0, 2.5, and 3.0), at a fixed NaOH concentration of 12 M. The 15 ambient-cured mixtures are evaluated for slump and mechanical performance at ambient temperature and after exposure to 300 °C. Complementary XRD, FTIR, TGA/DTG, FE-SEM, and EDX analyses of the representative FA50–MK50–SS/SH2.5 mixture support the interpretation of the reaction matrix and post-heating condition; they do not establish microstructural trends across all compositions.

The contribution is an integrated experimental and computational assessment of these composition–performance relationships within the investigated domain. Factorial analysis quantifies the separate and interactive effects of MK and SS/SH, while response-specific regression models with nested leave-one-out validation assess predictive performance and uncertainty. Lower-confidence-bound Pareto optimization identifies trade-offs between mechanical strength and thermal retention. Predictions are evaluated using three newly tested compositions and two independently reproduced design points. This framework provides locally evaluated candidate mixtures, but its interpretation is limited by the small dataset, the single exposure temperature, and the absence of a quantitative workability constraint in the optimization.

## 2. Materials and Methods

### 2.1. Experimental Studies

The binder comprised fly ash (FA), metakaolin (MK), and ground-granulated blast-furnace slag (GGBFS), activated with sodium hydroxide (NaOH) and sodium silicate (Na_2_SiO_3_) solutions. The fly ash was supplied by Medcem Cement, Mersin (Türkiye) and had a residue on the 45 µm sieve of approximately 15–30% and activity indices of ≥75% and ≥85% at 28 and 90 days, respectively [39]. GGBFS was obtained from OYAK Cement (Türkiye), with a density of 2.81 g/cm^3^ and a fineness of 4250 cm^2^/g [40]. The specified minimum Blaine fineness was 2750 cm^2^/g, while the specified minimum activity indices were 45% and 70% at 28 and 90 days, respectively. Metakaolin, supplied by AVS Mineral (İstanbul, Türkiye), had a median particle size (d_50_) of 1.35 µm (Sedigraph), an ISO brightness of 83%, and a pozzolanic activity of 1000 mg Ca(OH)_2_/g. The sodium silicate solution, supplied by Özkimsan (Bolu, Türkiye), had a silicate modulus (SiO_2_/Na_2_O) of 2.0. A 12 M NaOH solution was prepared by dissolving commercially available NaOH in water prior to geopolymer production. Table 1 provides the chemical compositions of the precursors.

**Figure 1:**
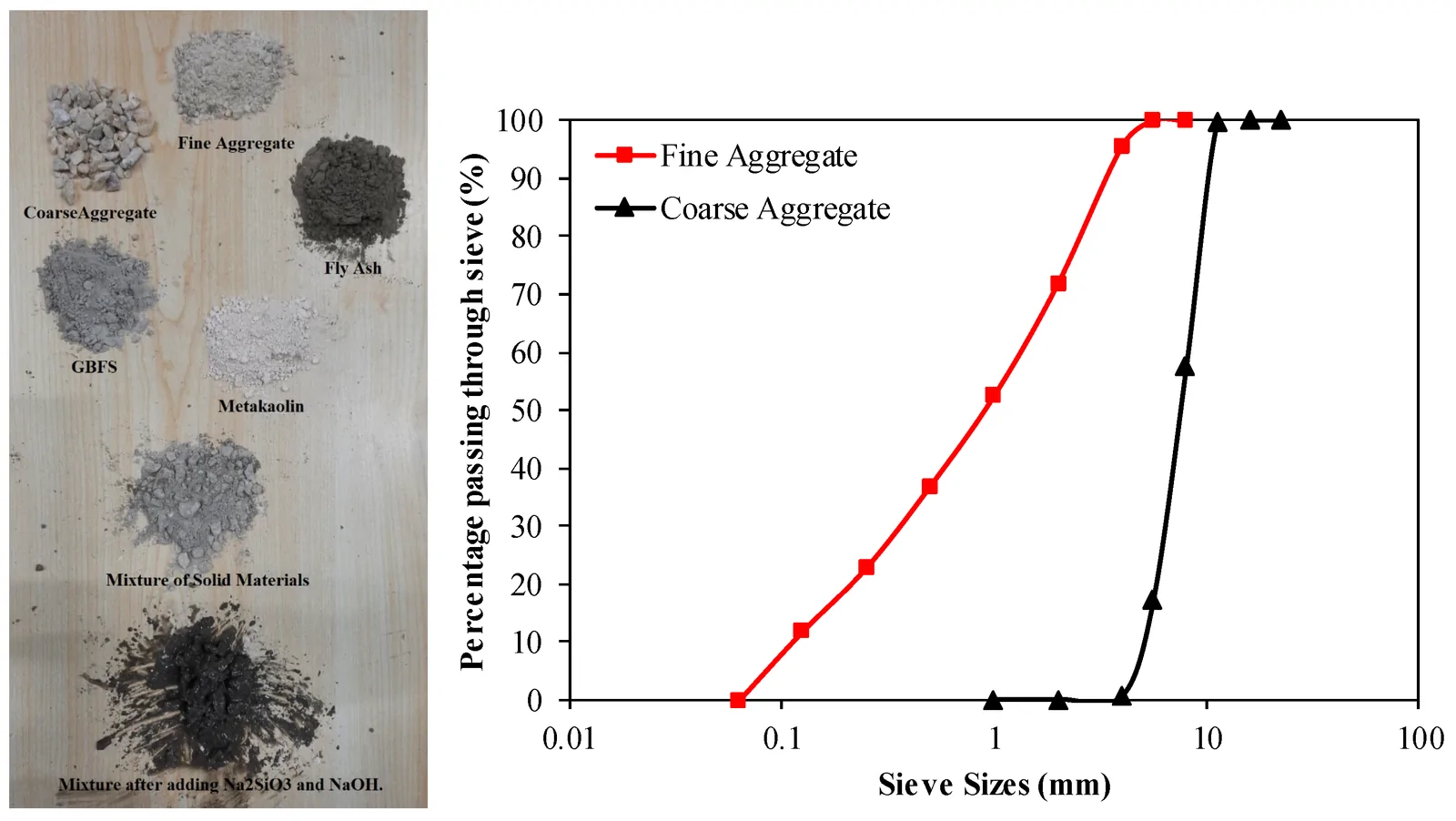
Materials used and particle size distribution of the aggregates.

Figure 1 shows the materials utilized in this study. Fine (0–4 mm) and coarse (4–11.2 mm) aggregates were used; their particle size distributions are given in Figure 1. Fifteen mixtures were prepared by combining three sodium silicate-to-sodium hydroxide (SS/SH) ratios (2.0, 2.5, and 3.0) with five MK replacement levels (0%, 25%, 50%, 75%, and 100% of the fly ash fraction). Table 2 presents the mixture proportions.

**Figure 2:**
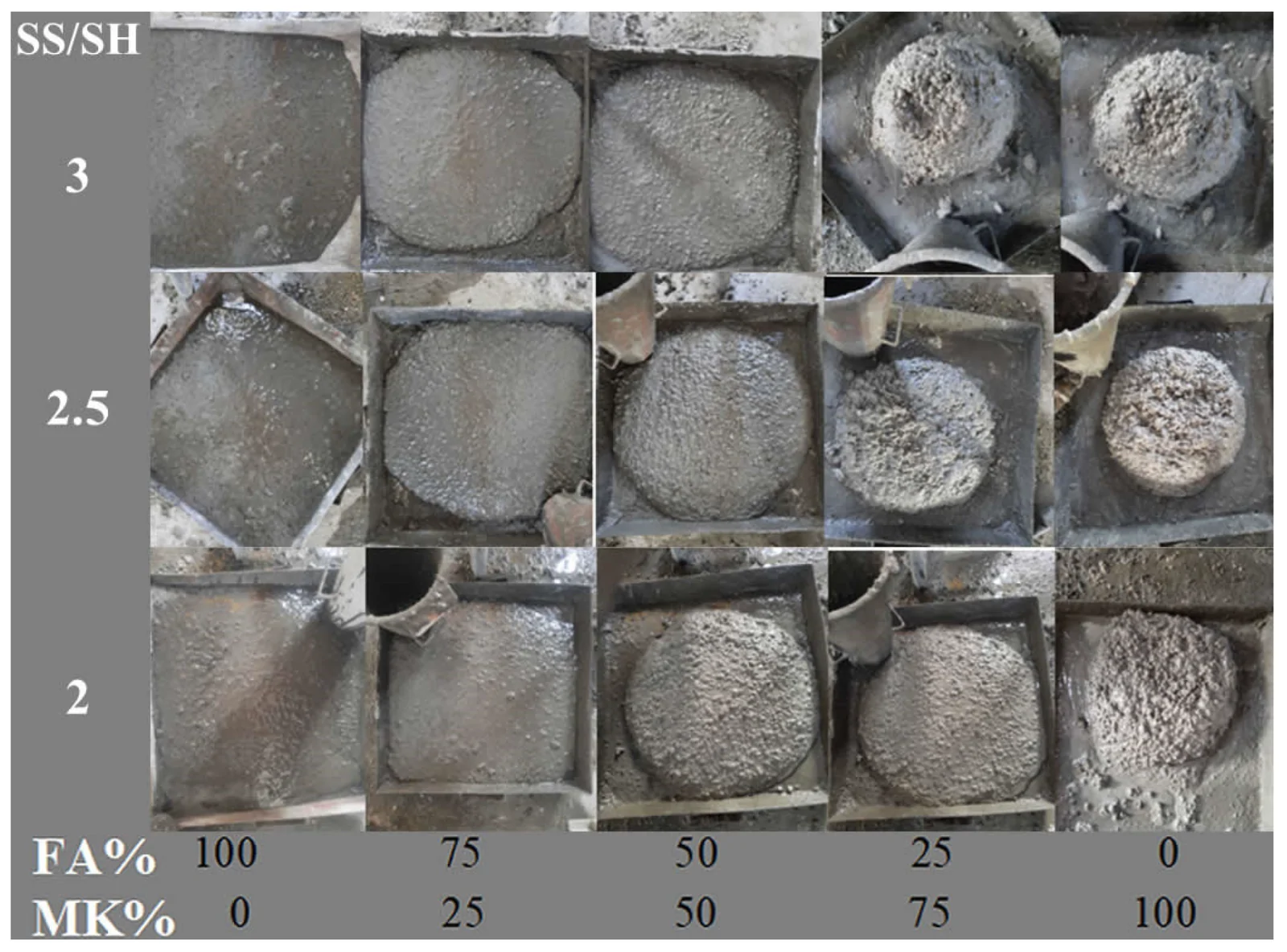

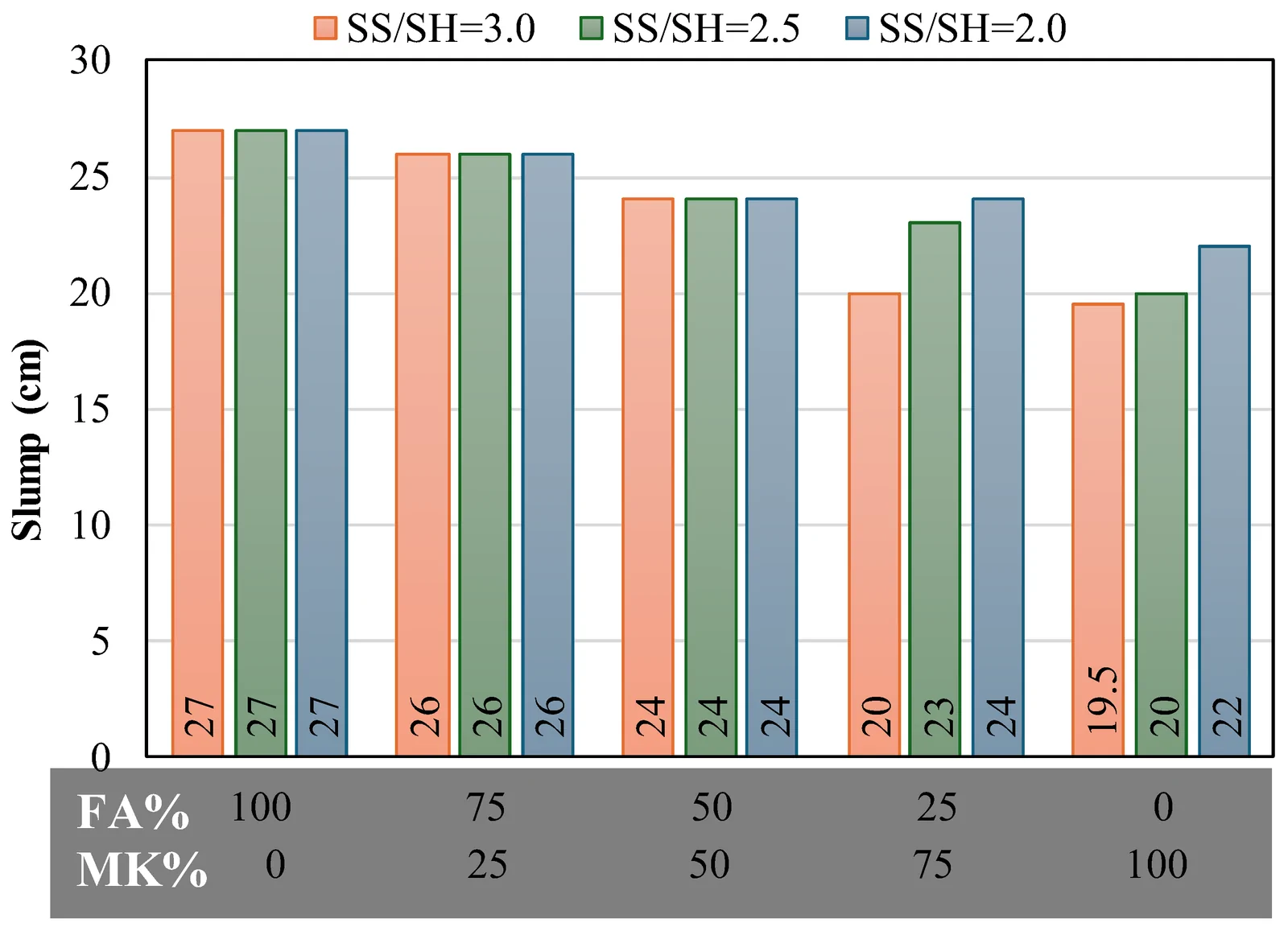
Slump test.

The alkali activator was prepared by mixing a 12 M NaOH solution with sodium silicate solution at the specified mass ratios. The NaOH solution was allowed to stand for approximately 24 h before use. Figure 2 presents the slump results: slump decreased with increasing MK replacement and with increasing SS/SH. Sunarsih et al. [41] also reported decreasing slump with increasing SS/SH in low-molarity fly ash–slag concrete. Hadi et al. [42] considered workability together with strength and setting time in ambient-cured geopolymer mix design. Hamed et al. [43] examined different alkaline activator combinations at SS/SH ratios of 2.0–3.0 and reported only small, inconsistent slump changes for NaOH-based activators, illustrating the need to interpret fresh-state behavior in its specific formulation context. FA–MK geopolymer studies provide additional relevant comparisons [44,45]. The FA–MK mortar study of Güneyisi and Gesoğlu [46] concerns a cement-containing system and is used only as a fresh-state comparison; it does not establish the reaction mechanisms of the present alkali-activated binder. The decrease in slump with both variables is consistent with reports that higher Na_2_SiO_3_/NaOH ratios and NaOH molarities reduce the workability of metakaolin geopolymers by increasing activator viscosity [47], and that MK contents of 30% or more increase paste viscosity and air entrapment in GGBS–MK geopolymer concrete [48].

**Figure 3:**
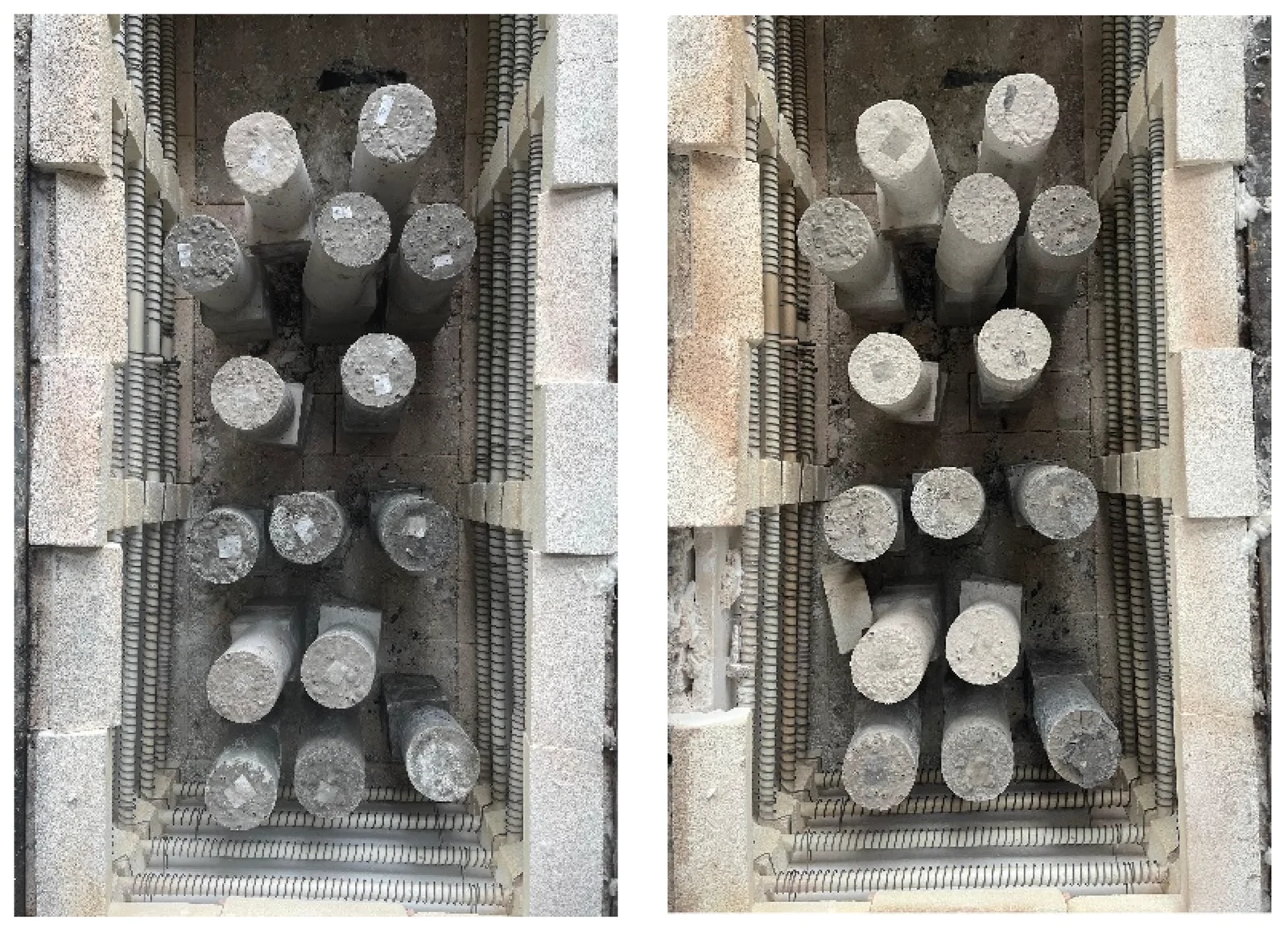
Specimens before and after exposure to 300 °C.

Test samples were prepared in 10 × 10 × 10-cm cubic molds and 10 × 20-cm cylindrical molds for each mixture series. All mixtures were produced using a laboratory-type mechanical mixer under the same mixing conditions. The samples were left in the mold for one day before curing. The samples were cured under ambient conditions. The samples were also exposed to an elevated temperature of 300 °C (Figure 3). The workability of the fresh geopolymer concrete mixtures was evaluated by the slump test in accordance with EN 12350-2 [49]. Compressive strength was determined at 28 days using 100 × 100 × 100 mm cubic specimens in accordance with EN 12390-3 [50]. Splitting tensile strength was determined using 100 × 200 mm cylindrical specimens in accordance with EN 12390-6 [51]. The specimens were centered in the testing machine and loaded continuously until failure. Compressive and splitting tensile strength tests were performed at 28 days. The specimens designated for elevated-temperature testing were exposed to 300 °C after completion of the 28-day curing period.

Alkali-activated materials comprise binders produced by reacting aluminosilicate or calcium-aluminosilicate precursors with alkaline activators [6,7]. The present binder contained FA and MK together with 125 kg/m^3^ GGBFS, equivalent to approximately 23.8% of the total precursor mass. FA and MK provide Si and Al, while GGBFS supplies Ca; this chemistry is compatible with an aluminosilicate reaction matrix containing calcium-bearing products. The term “hybrid geopolymer concrete” is used here within the broader category of alkali-activated materials. The XRD amorphous contribution, FTIR Si–O–T band, and EDX detection of Ca are consistent with this description, but do not uniquely identify or quantify separate N-A-S-H and C-(A)-S-H gel phases. Their possible coexistence is therefore an interpretation of the combined evidence rather than a phase-resolved measurement.

For physicochemical and microstructural characterization, the FA50–MK50–SS/SH2.5 mixture was selected as a representative midpoint composition rather than a maximum-strength or optimization-derived mixture. It contained equal FA and MK contents, an intermediate SS/SH ratio of 2.5, and the same GGBFS content used throughout the experimental program. This allowed the reaction products associated with the coexistence of FA, MK, and GGBFS to be examined without focusing on any single precursor end-member.

### 2.2. Statistical and Machine-Learning-Based Methodology

In the experimental design, the fifteen mixtures combined five metakaolin replacement levels (MK = 0, 25, 50, 75, and 100%) with three sodium silicate-to-sodium hydroxide ratios (SS/SH = 2.0, 2.5, and 3.0), and the corresponding fly ash content was calculated based on the mixture-closure constraint, which can be expressed as follows:

FA(%) = 100 − MK(%) (1)

Four mechanical responses were evaluated, all expressed in MPa: compressive strength at 24 °C (CS24), residual compressive strength after exposure to 300 °C (CS300), splitting tensile strength at 24 °C (STS24), and residual splitting tensile strength after exposure to 300 °C (STS300). The development dataset comprised 135 individual measurements: three replicate specimens per mixture for CS24 and two per mixture for each of the other responses. Replicate-level observations were retained for uncertainty estimation and factorial analysis, while mixture-level means were used as the response variables in the machine learning models.

Prior to any further analysis, the dataset was examined for duplicate mixture identifiers, label inconsistencies, missing observations, invalid factor combinations, and potential unit inconsistencies. Mixture identifiers were then reconstructed based on the actual FA, *MK*, and SS/SH values to ensure that each experimental composition was uniquely matched with its responses. Because FA and MK are perfectly correlated through the mixture closure constraint, FA was excluded from the machine-learning input matrix to avoid exact multicollinearity. The final predictor vector is as follows.

*x* = [MK,SS/SH] (2)

For each mixture–response combination, the arithmetic mean, sample standard deviation (with *n* − 1 degrees of freedom), standard error of the mean, coefficient of variation, and two-sided 95% confidence interval were computed. The sample standard deviation was calculated as follows:

(3) $$s=\sqrt{\frac{\sum_{i=1}^{n}{\left(y_{i}-\bar{y}\right)}^{2}}{n-1}}$$

where *y_i_* is the individual replicate measurement, $\bar{y}$ is the arithmetic mean of the replicate measurements, and *n* is the number of replicates. The coefficient of variation was calculated as

(4) $$\mathrm{CV}\left(\%\right)=100\;\frac{s}{\left|\bar{y}\right|}$$

and the 95% confidence interval of the experimental mean was obtained using the Student-t distribution:

(5) $${\mathrm{CI}}_{95\%}=\bar{y}\pm t_{0.975,\;n-1}\frac{s}{\sqrt{n}}$$

The retention of compressive and splitting tensile strength after thermal exposure was calculated for each mixture with the following formula:

(6) $$R_{\mathrm{CS}}\left(\%\right)=100\frac{{\mathrm{CS}}_{300}}{{\mathrm{CS}}_{24}}$$

(7) $$R_{\mathrm{STS}}\left(\%\right)=100\frac{{\mathrm{STS}}_{300}}{{\mathrm{STS}}_{24}}$$

Strength loss percentages corresponding to the above values were calculated as:

*L_CS_* = 100 − *R_CS_* (8)

*L_STS_* = 100 − *R_STS_* (9)

The equal-weight mean retention index is then:

(10) $$R_{\mathrm{mean}}=\frac{R_{\mathrm{CS}}+R_{\mathrm{STS}}}{2}$$

Ratio uncertainty was also assessed using first-order error propagation; however, because most of the post-heating responses were based on only two replicate specimens, the confidence intervals of the ratios were very sensitive to small-sample variance estimates. Consequently, the wide ratio intervals were retained only as diagnostic flags, not as the primary basis for model selection or robust optimization; in subsequent analyses, model-based predictive uncertainty was employed.

The relationships between mixture variables, mechanical properties, and thermal-retention metrics were analyzed at the mixture-mean level using Pearson product–moment and Spearman rank correlation analyses. Pearson product–moment correlation was used to evaluate linear relationships between the variables, while Spearman rank correlation was used to capture monotonic relationships without assuming linearity.

Two-way factorial analysis of variance was used to evaluate the main and interaction effects of MK and SS/SH on the four mechanical responses as follows:

*y_ijk_* = *μ* + *α_i_* + *β_j_* + (*αβ*)*_ij_* + *ε_ijk_* (11)

where *α_i_* represents the effect of MK level, *β_j_* represents the effect of SS/SH level, and (*αβ*)*_ij_* represents their interaction. Replicate-level data were used for the analysis, and the size of each effect was computed as partial eta squared, as follows:

(12) $${\eta_{p}}^{2}=\frac{{\mathrm{SS}}_{\mathrm{effect}}}{{\mathrm{SS}}_{\mathrm{effect}}+{\mathrm{SS}}_{\mathrm{error}}}$$

Separate regression models were developed for *CS*_24_, *CS*_300_, *STS*_24_ and *STS*_300_. The quadratic representation contained the following terms: MK, SS/SH, MK^2^, MK × SS/SH, (SS/SH)^2^. The general quadratic response function was utilized as

(13) $$\hat{y}=\beta_{0}+\beta_{1}\mathrm{MK}+\beta_{2}\left(\frac{\mathrm{SS}}{\mathrm{SH}}\right)+\beta_{3}{\mathrm{MK}}^{2}+\beta_{4}\mathrm{MK}\left(\frac{\mathrm{SS}}{\mathrm{SH}}\right)+\beta_{5}\;{\left(\frac{\mathrm{SS}}{\mathrm{SH}}\right)}^{2}$$

Ridge regularization was applied by minimizing:

(14) $$\sum_{i=1}^{n}{\left(y_{i}-\hat{y_{i}}\right)}^{2}+\tau \sum_{j=1}^{p}{\beta_{j}}^{2}$$

where *τ* is the regularization parameter, and to prevent data leakage, predictor scaling, construction of quadratic features, and the selection of the regularization parameter were performed only on the training data in each cross-validation fold.

Since there were only 15 independent mixture designs, we evaluated the regression models’ predictive performance using leave-one-out cross-validation (LOOCV). In each validation cycle, one mixture was withheld from model training and treated as an unseen test case, while the remaining 14 mixtures were used to train the model. The regularization parameter of the ridge regression models was chosen based only on the training portion of each fold, resulting in a nested LOOCV procedure with no information leakage. The performance metrics were calculated using the combined out-of-fold predictions over all LOOCV iterations, and model accuracy was evaluated in terms of the coefficient of determination (R^2^), root mean square error (RMSE), mean absolute error (MAE), range-normalized RMSE (NRMSE), mean bias (MB), and maximum absolute error (MxAE).

The primary criterion for selecting the best model was the lowest LOOCV RMSE; R^2^, MAE, NRMSE, mean bias, and maximum absolute error were used as auxiliary criteria to support model selection. Following this procedure, ridge-quadratic models were selected for CS_24_ and STS_24_, with regularization parameters of 0.01 and 0.0323, respectively; a homoscedastic GPR model was selected for CS_300_; and an unregularized quadratic regression model was selected for STS_300_.

Gaussian process regression (GPR) was also used for nonlinear probabilistic prediction with pointwise uncertainty estimates; its covariance structure was an anisotropic Matérn kernel with smoothness parameter ν = 2.5, combined with a white-noise kernel:

(15) $$k\left(x,x^{\prime }\right)=\sigma_{f}^{2}k_{\mathrm{Matérn},\;\nu =2.5}\left(x,x^{\prime }\right)+\sigma_{n}^{2}\delta_{x,x^{\prime }}$$

To account for the differing sensitivities of the two design variables, the model estimated separate length-scale parameters for MK and SS/SH and considered both homoscedastic and heteroscedastic GPR formulations. In the homoscedastic formulation, a single residual noise term was estimated for all observations, whereas in the heteroscedastic formulation, the response-specific experimental uncertainty obtained from the replicate statistics was incorporated into the model. Each GPR alternative was evaluated by LOOCV using the same held-out mixtures that the deterministic models used.

Probabilistic GPR models used for uncertainty propagation were chosen based on the response variable: a heteroscedastic GPR for CS24 and homoscedastic GPRs for CS300, STS24, and STS300. Pointwise 95% prediction intervals were calculated as follows:

*PI*_95(%)_ = *μ*(*x*) ± 1.96*σ*(*x*) (16)

where *μ*(*x*) and *σ*(*x*) are the GPR predictive mean and standard deviation, respectively.

Deterministic models were only used for the mechanical responses where they produced the lowest LOOCV prediction error, while the GPR surrogates were used for uncertainty-aware response mapping, robust optimization, and interval-based external validation since they can provide a consistent probabilistic description of all four mechanical responses (0 ≤ *MK* ≤ 100, 2.0 ≤ *SS*/*SH* ≤ 3.0).

No extrapolated mixture was included in the interpretation or optimization process, and all predictions were constrained within the experimentally defined design domain, screened to eliminate physically impossible negative strength values, and bounded within the range of the observed experimental response. For the GPR-based response surfaces, the local predictive standard deviations and the corresponding 95% prediction limits were also reported.

The number of independent experimental replicates depended on the response variable. For each of the 15 mixture designs, ambient-temperature compressive strength (CS24) was determined using three specimens (*n* = 3), while post-heating compressive strength (CS300), ambient-temperature splitting tensile strength (STS24), and post-heating splitting tensile strength (STS300) were each determined using two specimens (*n* = 2). Thus, the model development dataset comprised 45 individual CS24 measurements and 30 measurements each for CS300, STS24, and STS300. The response value used to fit the model was the arithmetic mean of the replicates at each mixture-design point, but the replicate-level observations were retained to estimate standard deviation, coefficient of variation, confidence intervals, and experimental uncertainty. For the external validation stage, the four mechanical responses were measured for each chosen mixture on three independently tested specimens (*n* = 3).

Model interpretation was performed using three complementary approaches. First, standardized quadratic ridge models were fitted separately for each response to compare the linear, quadratic, and interaction coefficients on equal terms; these models were used only for explanatory purposes (i.e., to assess the direction and relative importance of the response terms) and not as substitutes for the predictive models selected by LOOCV. Second, a global permutation sensitivity analysis was performed by independently permuting MK or SS/SH while keeping the other variable fixed, and the influence of each predictor was measured as the mean squared difference between the model’s original and permuted predictions. For each predictor–response pair, 500 permutations were performed, and the resulting feature contributions were normalized to sum to 100% for each mechanical response. Third, a variance-based Sobol sensitivity analysis was conducted within the bounded experimental-design domain. MK and SS/SH were treated as independent input variables sampled across the experimentally investigated ranges; first-order Sobol indices for MK and SS/SH were computed, and the remaining shared contribution was attributed to the second-order interaction between these two variables.

*S_MK_* + *S_SS_*_/*SH*_ + *S_interaction_* = 1 (17)

Total-order indices were calculated as

*S_T_*_,*MK*_ = *S_MK_* + *S_interaction_* (18)

*S_T_*_,*SS*/*SH*_ = *S_SS_*_/*SH*_ + *S_interaction_* (19)

The stability of the variance decomposition was evaluated using 1000 nonparametric bootstrap repetitions, and percentile-based 95% confidence intervals were subsequently calculated from the resulting bootstrap distributions.

Robust optimization was conducted over a discrete grid of 441 candidate mixtures, with MK varied from 0 to 100% in 5% increments and SS/SH varied from 2.0 to 3.0 in increments of 0.05. The corresponding FA content for each candidate mixture was calculated using the mixture closure constraint. For each candidate mixture, the GPR-based predictive mean, predictive standard deviation, and 95% lower and upper prediction bounds were obtained for each of the four mechanical responses. The conservative lower confidence bound was then defined as

*LCB_j_*(*x*) = *μ_j_*(*x*) − 1.96 *σ_j_*(*x*) (20)

and the upper bound was taken as

*UCB_j_*(*x*) = *μ_j_*(*x*) + 1.96 *σ_j_*(*x*) (21)

All predicted mean values and lower confidence bounds were assessed for physical admissibility prior to the optimization procedure. Three complementary performance metrics were then defined. The robust strength score was computed as the equal-weighted average of the min–max-normalized lower confidence bounds for the four mechanical responses:

(22) $$S_{\mathrm{strength}}=\frac{1}{4}\sum_{j=1}^{4}\tilde{{\mathrm{LCB}}_{j}}$$

Conservative compressive- and splitting-strength retention values were defined as

(23) $$R_{\mathrm{CS}}^{\mathrm{rob}}=100\;\frac{{\mathrm{LCB}}_{\mathrm{CS}300}}{{\mathrm{UCB}}_{\mathrm{CS}24}}$$

(24) $$R_{\mathrm{STS}}^{\mathrm{rob}}=100\;\frac{{\mathrm{LCB}}_{\mathrm{STS}300}}{{\mathrm{UCB}}_{\mathrm{STS}24}}$$

The balanced robust-retention score was then calculated as

(25) $$S_{\mathrm{retention}}=\frac{{\tilde{R}}_{\mathrm{CS}}^{\mathrm{rob}}+{\tilde{R}}_{\mathrm{STS}}^{\mathrm{rob}}}{2}$$

The reliability score is computed using the inverse min–max normalization of the mean relative GPR uncertainty, where relative uncertainty is defined as

*σ_j_*/*μ_j_* (26)

Pareto optimality was determined by simultaneously maximizing the robust strength score and the balanced robust-retention score. A candidate mixture was considered Pareto optimal if no other candidate mixture performed at least as well as the current candidate in both objectives and strictly better in at least one. The compromise solution was identified by the minimum Euclidean distance to the ideal point in normalized two-objective space. The term “knee-point candidate” refers to this distance-to-ideal compromise and does not imply a separate curvature-based knee calculation. The mixtures corresponding to the maximum robust strength, maximum robust retention, and reliable high-strength performance were also retained for experimental evaluation to represent different positions along the Pareto front.

Five mixtures were selected for external experimental validation: the FA50–MK50–SS/SH2.50 reference mixture and four optimization-derived mixtures representing the knee point, maximum robust retention, reliable high strength, and maximum robust strength. Each response was measured using three independently tested specimens per mixture, yielding 60 individual measurements and 20 mixture–response records. Experimental means, sample standard deviations, and coefficients of variation were calculated. Validation observations were excluded from model fitting, hyperparameter tuning, and model reselection.

The external residual was determined as

(27) $$e_{i}=y_{i,\mathrm{measured}}-{\hat{y}}_{i,\mathrm{predicted}}$$

The prediction residual was defined such that a positive value indicates that the model underpredicts. External predictive performance was evaluated separately for each mechanical response using R^2^, RMSE, MAE, MAPE, NRMSE, mean bias, and maximum absolute error. Because only five validation mixtures were available for each response, these response-specific performance metrics were interpreted descriptively rather than as definitive statistical measures. Prediction interval coverage was evaluated using two criteria: the strict coverage criterion, which required that the experimental mean fall within the original 95% GPR prediction interval; and a secondary, resolution-adjusted coverage criterion, in which the lower and upper interval limits were expanded by half the measurement resolution because the experimental measurements were reported to a resolution of 0.01 MPa.

∆*_resolution_*= ±0.005 MPa (28)

A validation record was considered valid if three experimental measurements were available, the measured mean fell within the prediction-interval criterion, the absolute relative error did not exceed 10%, and the experimental CV was less than 10%. Results that were within the resolution-adjusted prediction interval, or within 0.005 MPa of an interval boundary, were considered to be borderline validated. Records outside the adjusted interval were not discarded as outliers but retained in the dataset and reported for further evaluation. Parity diagrams were used to compare predicted and measured responses; GPR prediction intervals were represented as horizontal error bars, and experimental standard deviations as vertical error bars. Residual and relative-error plots were also examined to determine the direction of prediction errors, identify local deviations, and verify that each validation record met the a priori 10% relative-error limit.

## 3. Results and Discussion

### 3.1. Compressive Strength

**Figure 4:**
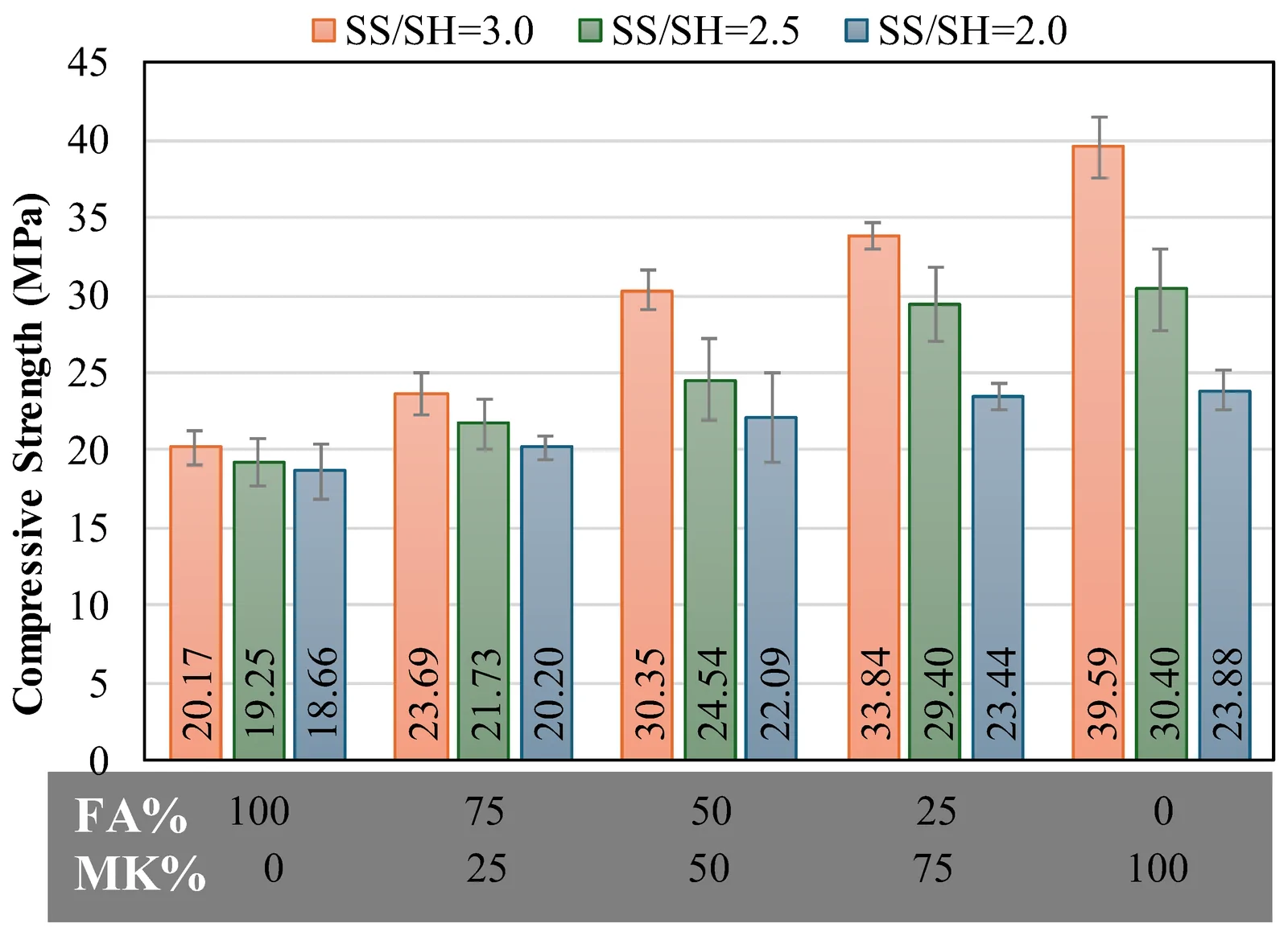
Compressive strength of the samples.

**Figure 5:**
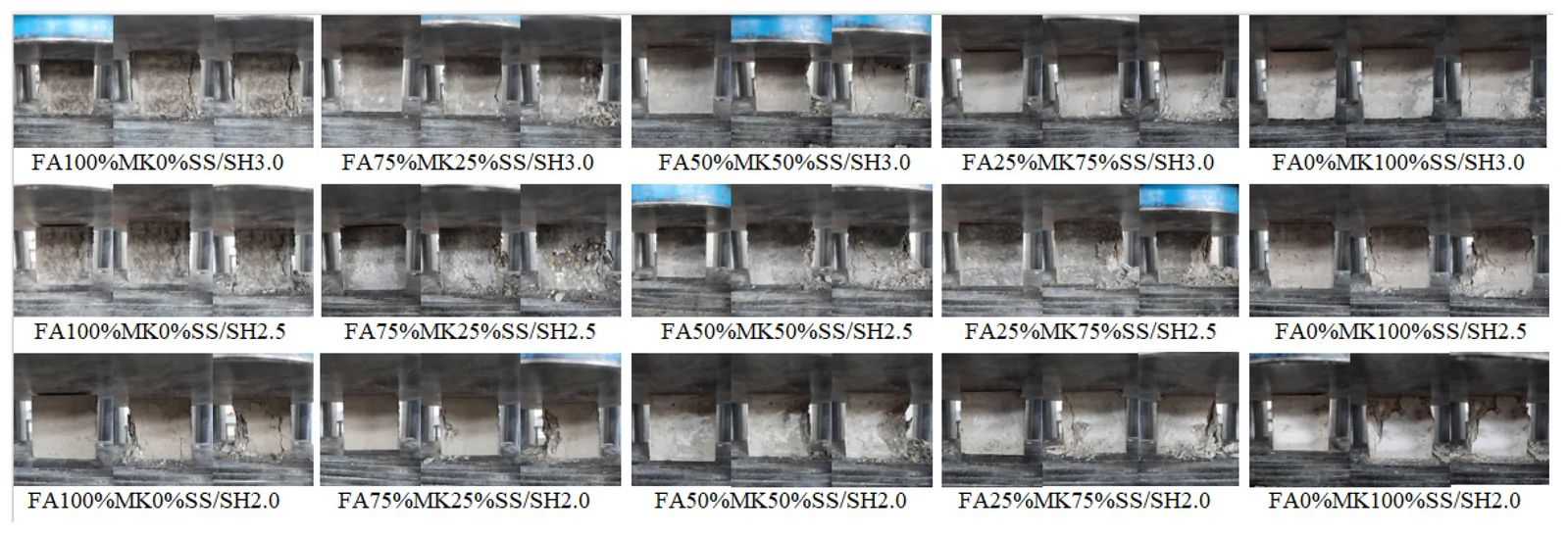
Failure of the samples under compression.

Figure 4 and Figure 5 present the ambient compressive strengths and specimen failure patterns. Both MK replacement and SS/SH influenced the compressive strength. The 15 mixtures were prepared by combining MK replacement levels of 0%, 25%, 50%, 75%, and 100% with SS/SH ratios of 2.0, 2.5, and 3.0. Because the slump decreased as either of the variables increased, the strength results should be considered alongside the associated reduction in workability.

In the SS/SH = 3.0 series, compressive strength increased gradually and significantly as the metakaolin content rose. The compressive strength, which was 20.17 MPa in the mixture containing 0% MK, reached 23.69 MPa at the 25% MK level, showing an increase of 17.47%. When the MK content was increased to 50%, the strength reached 30.35 MPa, representing an increase of 50.48% compared to the initial mixture. At a 75% MK level, the strength reached 33.84 MPa, representing a 67.75% increase. The highest value, 39.59 MPa, was measured in the mixture containing 100% MK, representing a total increase of 96.27%. In this series, the effect of metakaolin was most pronounced.

In the SS/SH = 2.5 series, higher metakaolin content also consistently increased compressive strength. The strength of the reference mixture containing 0% MK was 19.25 MPa; it rose to 21.73 MPa at 25% MK content, representing a 12.86% increase. At a 50% MK ratio, the strength reached 24.54 MPa, representing a 27.49% increase. At the 75% MK level, the strength reached 29.40 MPa, representing a 52.70% increase. At 100% MK usage, the compressive strength reached 30.40 MPa, with a total increase of 57.93%. These results indicate that metakaolin significantly increased the strength at an SS/SH ratio of 2.5, although this increase was smaller than that in the SS/SH = 3.0 series.

Although the positive effect of metakaolin addition persisted in the SS/SH = 2.0 series, strength increases remained lower than in the other series. The compressive strength, which was 18.66 MPa in the mixture containing 0% MK, rose to 20.20 MPa at the 25% MK level, showing an increase of 8.27%. At an MK content of 50%, the strength reached 22.09 MPa, representing an increase of 18.35%. At the 75% MK level, a strength of 23.44 MPa was achieved, representing a 25.59% increase. In the 100% MK mixture, the strength reached 23.88 MPa, representing a total increase of 27.98%. This indicates that the contribution of metakaolin persists at a low SS/SH ratio, but any increases are smaller owing to the limiting effect of the activator composition.

When the series were evaluated together, the highest compressive strengths for the same MK ratio were generally obtained in the SS/SH = 3.0 mixtures. The response to SS/SH is formulation-dependent: Morsy et al. [32] and Niş [35] investigated different fly ash-based systems, so the present increase over SS/SH = 2.0–3.0 should not be generalized beyond this mixture domain. Recent FA–MK studies support this restriction: in ambient-cured MK–FA geopolymer concrete without slag, Khatri et al. [52] identified an MK:FA ratio of 60:40 and an Na_2_SiO_3_/NaOH ratio of 2.5 as optimum, with lower strengths at higher MK contents and higher ratios, whereas Boonmema et al. [53] obtained the highest strengths of heat-cured MK–FA geopolymer mortars at MK:FA = 30:70. The monotonic increases observed here up to 100% MK and SS/SH = 3.0 therefore reflect the present slag-containing formulation and activator dosage rather than a general optimum. Additionally, this series exhibited the highest sensitivity to increases in metakaolin. Indeed, at the 25% MK level, the increases were 17.47%, 12.86%, and 8.27% in the SS/SH = 3.0, 2.5, and 2.0 series, respectively. Similarly, the total increases at the 100% MK level were 96.27%, 57.93%, and 27.98%, respectively. These findings indicate that the effectiveness of the metakaolin additive is more pronounced at higher SS/SH ratios and that the reactive structure provided by metakaolin is utilized more efficiently in an environment with sufficient silicate. This interpretation agrees with Abdeen et al. [47], who found that the compressive strength of MK geopolymers depended more strongly on the soluble SiO_2_ supplied by Na_2_SiO_3_ than on the total SiO_2_ content of the metakaolin.

**Figure 6:**
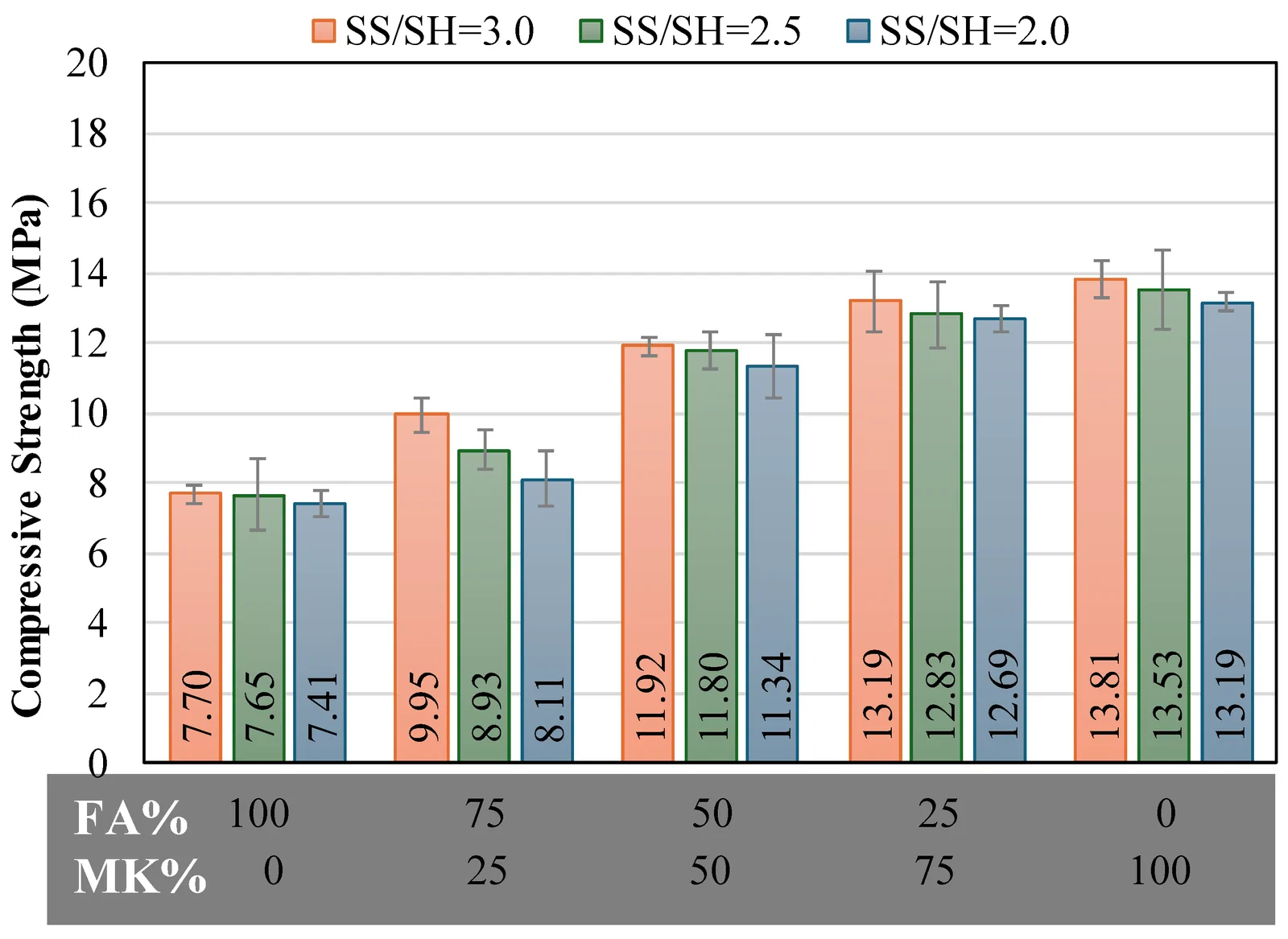
Residual compressive strength of the mixtures after exposure to 300 °C.

**Figure 7:**
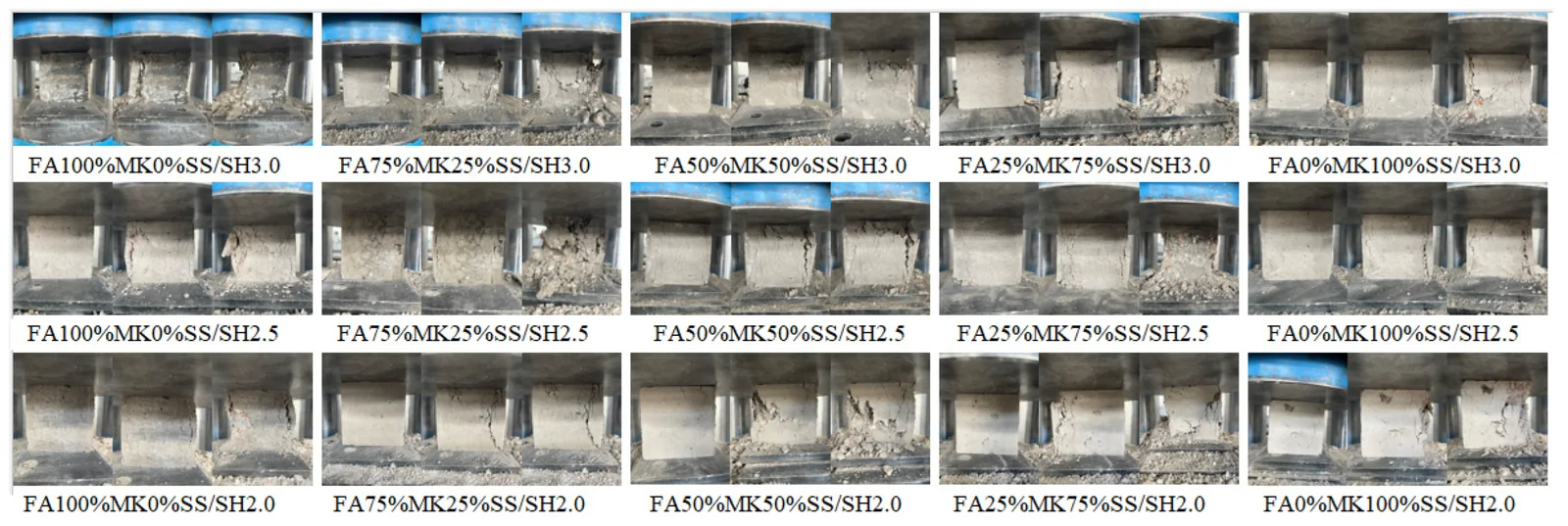
Compressive failure patterns of the specimens after exposure to 300 °C.

The compressive strength results of the samples exposed to 300 °C are presented in Figure 6. The observed failures are depicted in Figure 7. In the SS/SH = 3.0 series, the compressive strength of the samples exposed to 300 °C increased steadily with higher metakaolin content. The strength increased from 7.70 MPa in the mixture containing 0% MK to 9.95 MPa at a 25% MK ratio, representing a 29.25% increase. At the 50% MK level, the compressive strength reached 11.92 MPa, representing an increase of 54.76%. In the mixture containing 75% MK, the value reached 13.19 MPa, representing a 71.34% increase. The mixture containing 100% MK yielded the highest value, 13.81 MPa, corresponding to a total increase of 79.31%.

In the SS/SH = 2.5 series, increasing metakaolin content consistently increased the residual compressive strength after exposure to 300 °C. The strength of the reference mixture (0% MK) was 7.65 MPa and increased to 8.93 MPa at 25% MK, representing a 16.75% increase. At a 50% MK ratio, the value reached 11.80 MPa, representing an increase of 54.23%. At a 75% MK level, the residual strength reached 12.83 MPa, representing a 67.72% increase. In the mixture containing 100% MK, the value reached 13.53 MPa, representing a total increase of 76.89%.

The same trend was observed in the SS/SH = 2.0 series. The post-300 °C strength increased from 7.41 MPa in the mixture containing 0% MK to 8.11 MPa at the 25% MK level, representing a 9.49% increase. At a 50% MK ratio, the residual compressive strength reached 11.34 MPa, with an increase of 53.02%. The mixture containing 75% MK achieved 12.69 MPa, representing a 71.28% increase. In the mixture containing 100% MK, the residual compressive strength reached 13.19 MPa, representing a total increase of 77.94%.

The reduction in compressive strength after exposure to 300 °C is consistent with water loss, dehydration of reaction products, and thermally induced strain incompatibility. For the representative post-heating sample, TGA/DTG showed further low-temperature water loss and a broader dehydration region. Because this material had already been heated before characterization, these curves cannot quantify the water lost during the original exposure or demonstrate its direct causal contribution to strength loss. A plausible mechanism is that dehydration-related contraction of the reaction matrix, combined with different thermal strains in aggregates and residual particles, promotes local matrix cracking and interfacial stress concentrations. Such defects reduce the effective load-bearing area after cooling. The thermal study of Özbayrak et al. [54] likewise links activator-dependent thermal behavior with microstructural changes. Carbonate decomposition observed well above 300 °C is not a plausible principal cause of the strength losses measured at 300 °C.

Differences between absolute residual strength and proportional retention also reflect the interaction between MK and SS/SH. At SS/SH = 2.0, increasing MK replacement from 0% to 100% increased the ambient compressive strength from 18.66 to 23.88 MPa (+27.98%) and the residual strength from 7.41 to 13.19 MPa (+77.94%). Consequently, retention increased from 39.71% to 55.23%, whereas strength loss decreased from 60.29% to 44.77%. Replacing FA with MK may increase the availability of dissolved Al- and Si-bearing species, thereby promoting a more continuous reaction matrix. A higher hydroxide fraction at lower SS/SH may favor MK dissolution, while a lower soluble-silicate fraction may alter shrinkage sensitivity and moisture transport. A dependence of the MK benefit on alkali availability has also been reported for FA–MK geopolymer aggregates, in which water absorption increased and granule strength decreased when MK exceeded 20 wt% because the alkali content was insufficient [55]. These are possible mechanisms, rather than composition-wide microstructural observations. At 100% MK, an SS/SH ratio of 3.0 produced a much higher ambient strength of 39.59 MPa. However, across all three SS/SH ratios residual strengths at 100% MK were similar (13.19–13.81 MPa), whereas ambient strengths ranged from 23.88 to 39.59 MPa. The higher retention at SS/SH = 2.0 therefore reflects both the thermal response and the ratio-based definition of retention; it does not indicate the highest absolute residual strength.

Across the three SS/SH series, residual compressive strength increased with MK replacement. The relative gains at 50% MK were similar (54.76%, 54.23%, and 53.02% for SS/SH values of 3.0, 2.5, and 2.0, respectively). At 75% MK, the corresponding gains were 71.34%, 67.72%, and 71.28%, and at 100% MK, they were 79.31%, 76.89%, and 77.94%. The influence of activator ratio on the relative benefit of MK was thus less pronounced after heating. At 100% MK, the relative gains in ambient and residual compressive strengths were 96.27% and 79.31% at SS/SH = 3.0, 57.93% and 76.89% at SS/SH = 2.5, and 27.98% and 77.94% at SS/SH = 2.0, respectively. These comparisons use the corresponding MK-free mixture under each thermal condition as the reference. Exposure to 300 °C reduced the compressive strength of every mixture. At MK replacement levels of 0%, 25%, 50%, 75%, and 100%, the losses were 61.82%, 58.00%, 60.72%, 61.02%, and 65.12% for SS/SH = 3.0; 60.26%, 58.90%, 51.92%, 56.36%, and 55.49% for SS/SH = 2.5; and 60.29%, 59.85%, 48.64%, 45.86%, and 44.77% for SS/SH = 2.0. Although increasing MK improved absolute residual strength in all series, it consistently reduced proportional strength loss only at SS/SH = 2.0. High MK content, therefore, did not confer the same retention benefit at every activator ratio.

The reduced SS/SH separation after heating is particularly clear at 100% MK: the ambient compressive strengths span 23.88–39.59 MPa, whereas the residual values span only 13.19–13.81 MPa. A higher SS/SH ratio can favor initial matrix development through the balance of soluble silicate and hydroxide available for precursor reaction. Heating introduces additional controls—water removal, matrix contraction, and strain mismatch—that can diminish this initial advantage. At high MK replacement, the common precursor skeleton and fixed slag content may therefore become more influential than the initial activator ratio. This explanation is consistent with the smaller, statistically non-significant SS/SH effect on CS300 after multiplicity adjustment (Section 5), but it does not demonstrate identical post-heating matrices. Moreover, changing SS/SH changes the proportions of two activator solutions; it cannot be interpreted as an isolated change in bulk Si/Al or Na_2_O content without the full solution compositions. Özbayrak et al. [54] similarly reported that, in fly ash geopolymer concrete, a higher SS/SH ratio increased the ambient compressive strength but had no clear effect on the compressive strength after exposure to 400–800 °C. A recent mechanistic comparison is provided by Barzoki and Gowayed [56], who varied silica/alumina composition in MK geopolymers and reported competing effects of dehydration and temperature-dependent reaction processes. Their silica-rich and alumina-rich formulations did not maximize the same mechanical and thermal responses. This supports treating initial strength and post-heating stability as separate objectives. However, their heat-cured, calcium-poor formulations and flexural measurements differ from the present ambient-cured FA–MK–slag concrete. Their results support a chemistry-dependent interpretation, rather than a quantitative prediction of the losses measured here or proof of secondary geopolymerization at 300 °C.

### 3.2. Splitting Tensile Strength

**Figure 8:**
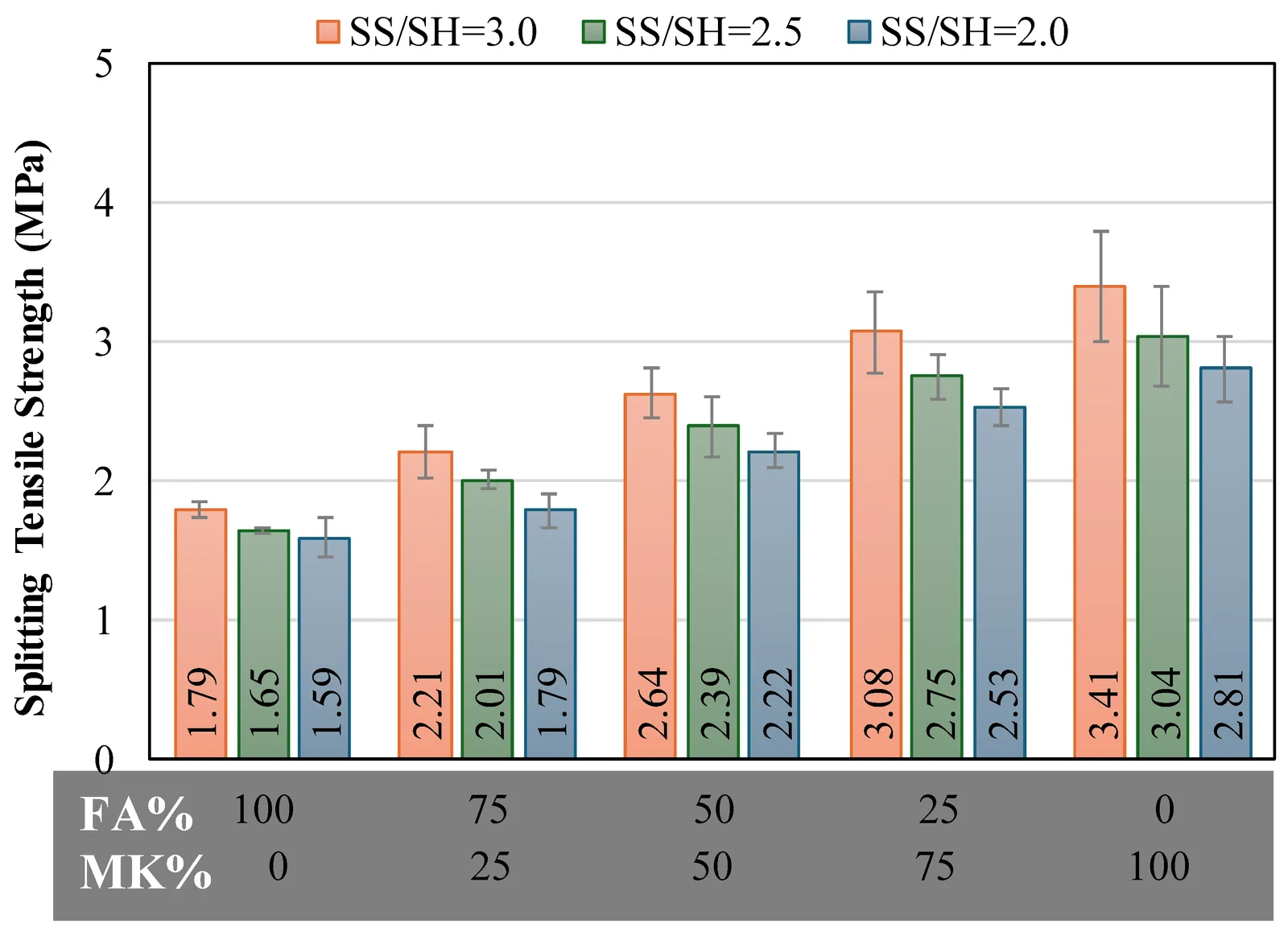
Splitting tensile strength of the samples.

Splitting tensile strength results indicate that as the metakaolin (MK) content increases, tensile strength at 24 °C rises consistently across all SS/SH series (Figure 8). This increase is consistent with the compressive strength results and suggests improved matrix continuity and stress transfer. Additionally, absolute strength values increase with the SS/SH ratio, but the rate of increase varies with MK content.

In the SS/SH = 3.0 series, increasing the metakaolin ratio had a particularly pronounced effect on splitting tensile strength. The strength of the mixture containing 0% MK was 1.79 MPa and increased to 2.21 MPa at 25% MK (a 23.19% increase), to 2.64 MPa at 50% MK (a 47.25% increase), to 3.08 MPa at 75% MK (a 71.87% increase), and to 3.41 MPa at 100% MK (a 90.25% increase). This result establishes an increase in splitting tensile capacity; it is consistent with improved matrix stress transfer, but the test does not independently quantify crack-growth resistance or aggregate–binder bond strength.

A similar trend was observed in the SS/SH = 2.5 series. The strength in the mixture containing 0% MK was 1.65 MPa and increased to 2.01 MPa at 25% MK (a 21.97% increase), to 2.39 MPa at 50% MK (a 44.99% increase), to 2.75 MPa at 75% MK (a 66.69% increase), and to 3.04 MPa at 100% MK (an 84.31% increase). These results demonstrate that the effect of metakaolin on tensile strength persists and is systematic across different SS/SH ratios.

Although the initial strengths are lower in the SS/SH = 2.0 series, the effect of metakaolin addition remains evident. The strength, which was 1.59 MPa at 0% MK, increased by 12.60% (to 1.79 MPa) at 25% MK, by 39.44% (to 2.22 MPa) at 50% MK, by 59.10% (to 2.53 MPa) at 75% MK, and by 76.66% (to 2.81 MPa) at 100% MK. This result demonstrates that metakaolin improves tensile behavior even at low SS/SH ratios.

**Figure 9:**
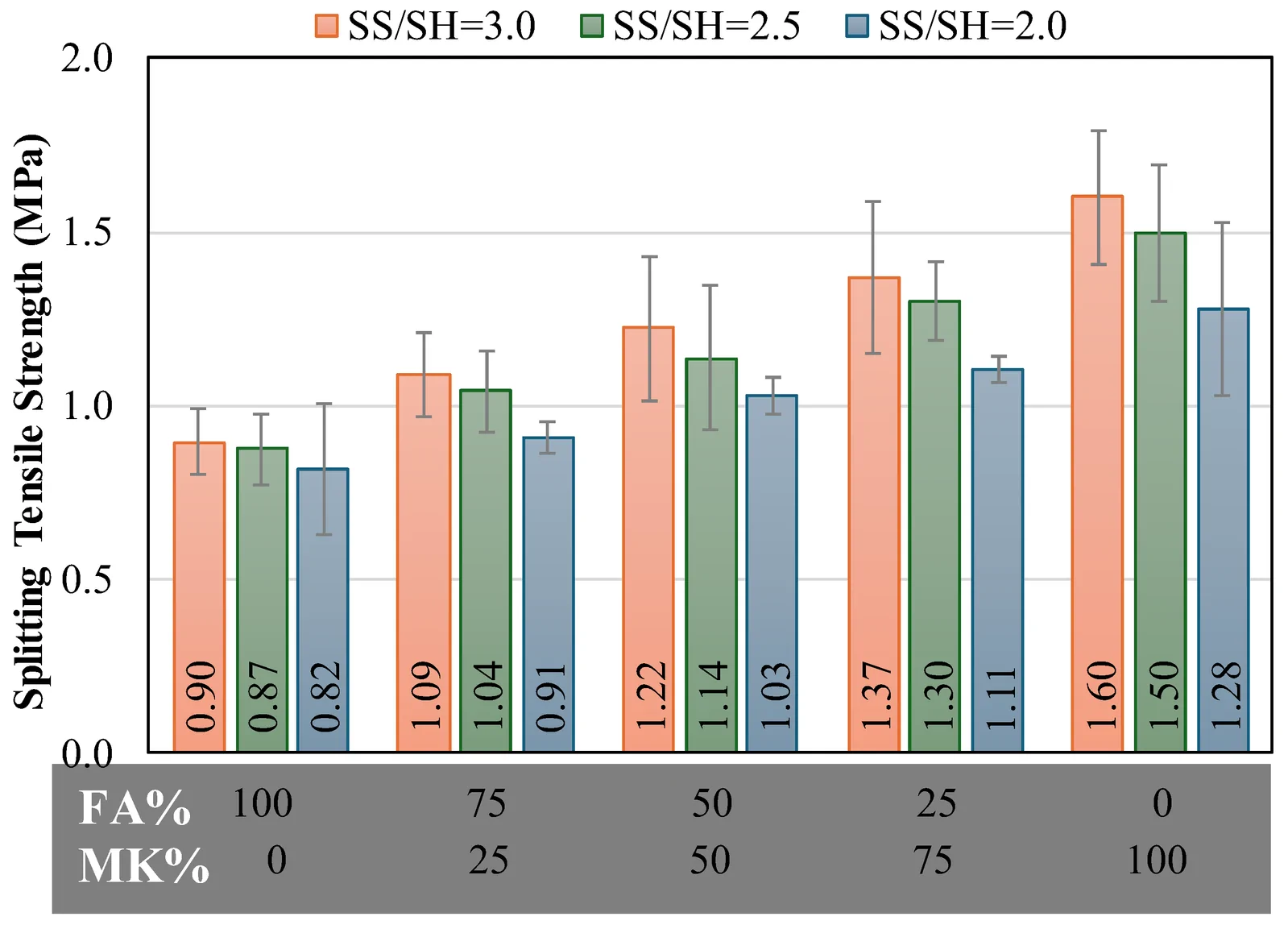
Residual splitting tensile strength of the mixtures after exposure to 300 °C.

**Figure 10:**
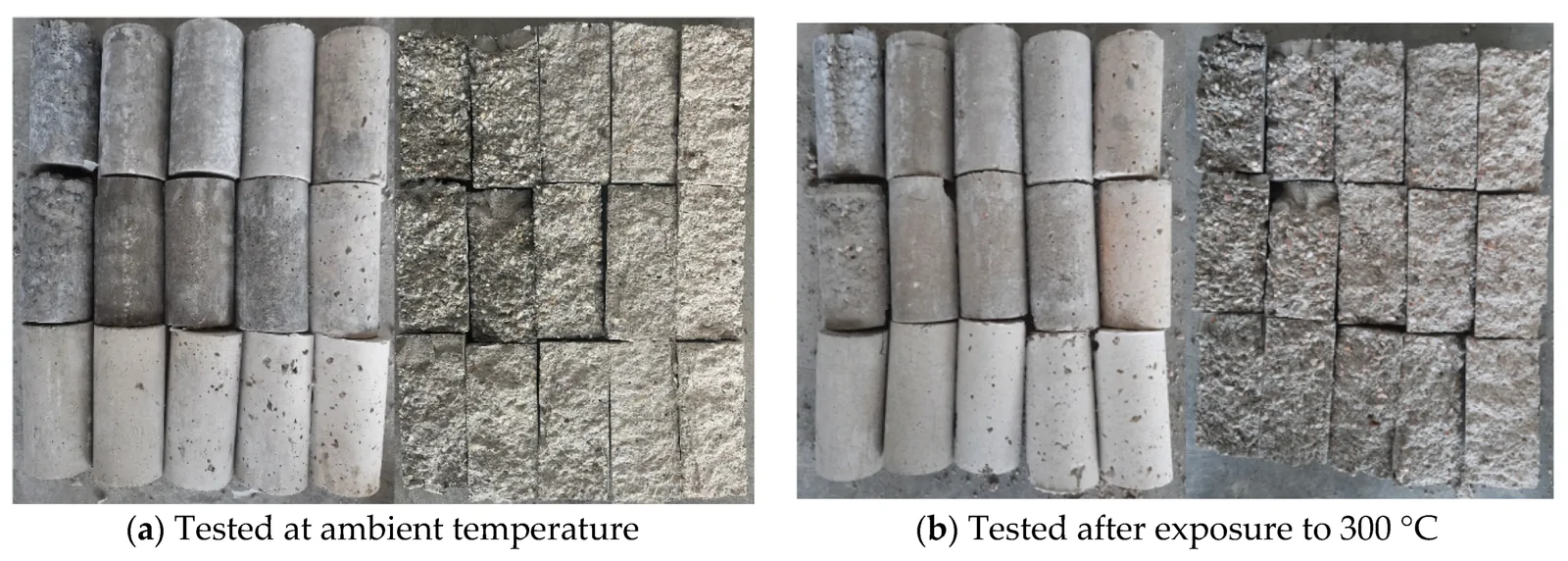
Cylindrical specimens.

The effect of metakaolin persists after heating to 300 °C (Figure 9). The samples are shown in Figure 10. In the SS/SH = 3.0 series, the residual strength was 0.90 MPa at 0% MK and increased by 20.85% (to 1.09 MPa) at 25% MK; by 35.80% (to 1.22 MPa) at 50% MK; by 51.91% (to 1.37 MPa) at 75% MK; and by 77.76% (to 1.60 MPa) at 100% MK. This indicates that MK replacement also increased the absolute residual tensile strength after exposure to 300 °C.

In the SS/SH = 2.5 series, the strength of 0.87 MPa at 0% MK increased to 1.04 MPa (19.63%) at 25% MK, to 1.14 MPa (30.72%) at 50% MK, to 1.30 MPa (49.46%) at 75% MK, and to 1.50 MPa (71.94%) at 100% MK. These results show that MK also increased the absolute residual tensile strength at the intermediate SS/SH ratio.

In the SS/SH = 2.0 series, the strength of 0.82 MPa at 0% MK increased by 10.72% to 0.91 MPa at 25% MK, by 25.47% to 1.03 MPa at 50% MK, by 34.76% to 1.11 MPa at 75% MK, and by 55.66% to 1.28 MPa at 100% MK. Although the increase continued in this series, it remained less pronounced than in the other series.

The post-heating tensile response also reflects damage to stress-transfer paths. Local matrix cracks and debonded interfaces can act as preferential fracture paths under splitting, while pores and incompletely reacted particle boundaries can concentrate tensile stress. These mechanisms help explain why greater absolute tensile strength does not ensure greater proportional retention. At 100% MK, changing SS/SH from 2.0 to 3.0 increases STS24 from 2.81 to 3.41 MPa and STS300 from 1.28 to 1.60 MPa. Thus, an activator-related difference persists after heating; the convergence is less marked than for compressive strength. The representative SEM observations support the presence of local defects but, without matched unheated images and quantitative crack measurements, cannot establish how much of the tensile loss was caused by newly formed thermal cracks.

## 4. Physicochemical and Microstructural Characterization

All microstructural, mineralogical, spectroscopic, and thermal characterization analyses were performed at the Technology Research and Application Center (TAUM), Erciyes University, Kayseri, Türkiye. The characterized specimens were cured for 28 days and exposed to 300 °C before analysis by XRD, FTIR, TGA/DTG, FE-SEM, and EDX.

### 4.1. X-Ray Diffraction (XRD) Analysis

**Figure 11:**
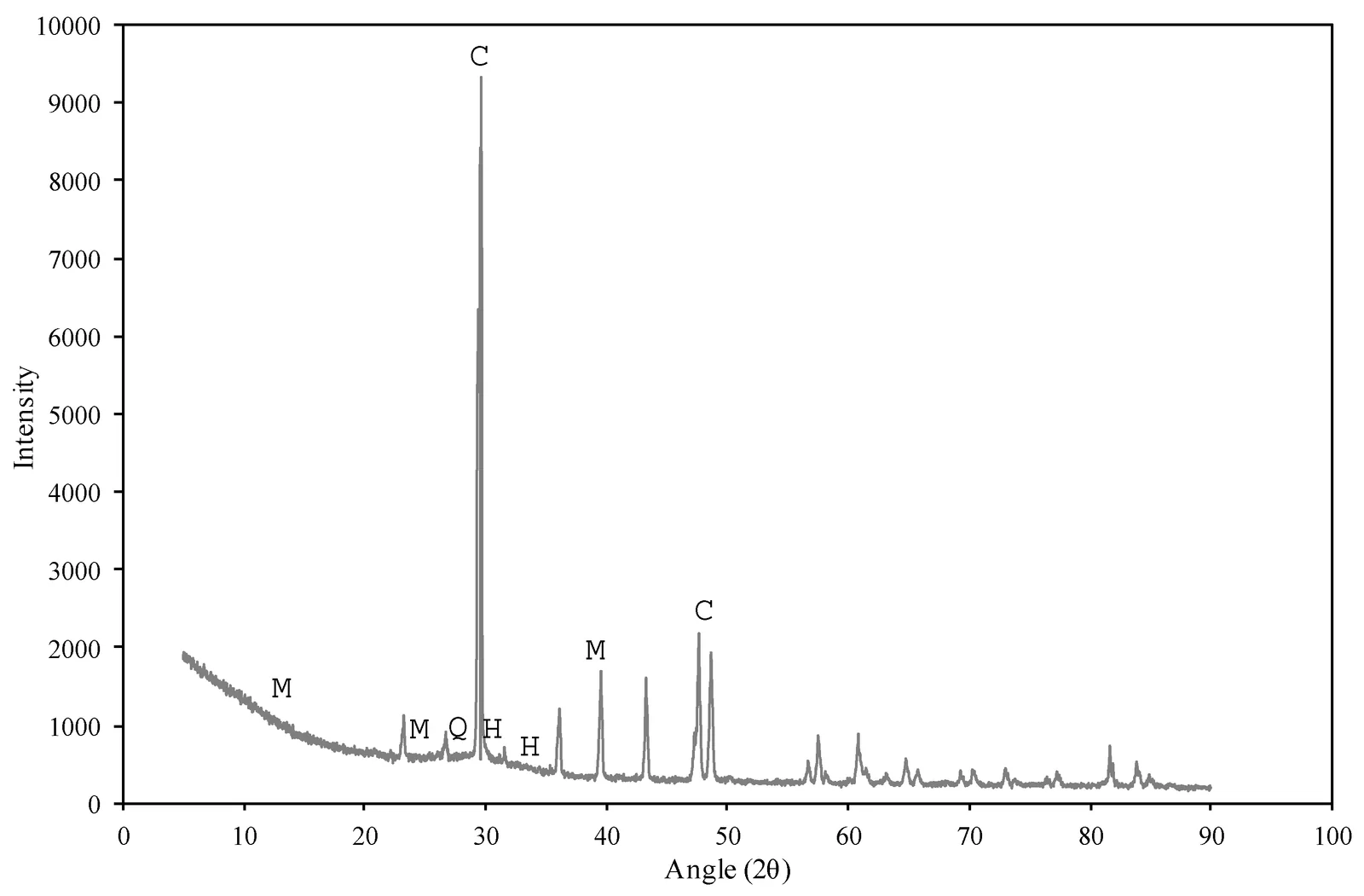
XRD pattern of the representative FA50–MK50–SS/SH2.5 mixture. C: calcite; Q: quartz; M: mullite; H: hematite.

The XRD pattern of the representative FA50–MK50–SS/SH2.5 mixture exhibited a broad, diffuse halo at approximately 2θ = 20–35° (Figure 11), indicating a predominantly amorphous or poorly crystalline character coexisting with the identified crystalline reflections. This feature is consistent with alkali-activated aluminosilicate reaction products formed from FA, MK, and the activator. Calcium supplied by GGBFS may also contribute to the formation of calcium-containing products. However, the broad halo does not permit individual identification or quantitative separation of N-A-S-H and C-(A)-S-H gel phases by XRD alone.

In addition to the amorphous background, several crystalline reflections were observed. The reflection around 2θ ≈ 26.6° can be associated mainly with quartz, while reflections observed at approximately 21°, 36°, and other related positions are also consistent with quartz-containing crystalline phases. Peaks around 16°, 25°, 33°, and 40° may be associated with mullite, and reflections attributable to hematite were also observed. A distinct reflection at approximately 29.4–30° is consistent with the presence of calcite or other carbonate-containing crystalline phases.

The XRD pattern indicates the coexistence of residual crystalline phases and a predominantly amorphous matrix. Low crystallinity and overlapping reflections prevent conclusive assignment of individual reaction products. The results were therefore interpreted in conjunction with FTIR, TGA/DTG, FE-SEM, and EDX observations. The reflection near 2θ = 29.4–30° is consistent with calcite (CaCO_3_), potentially associated with carbonation; it does not independently establish the presence of C-(A)-S-H gel.

### 4.2. Fourier Transform Infrared Spectroscopy (FTIR) Analysis

**Figure 12:**
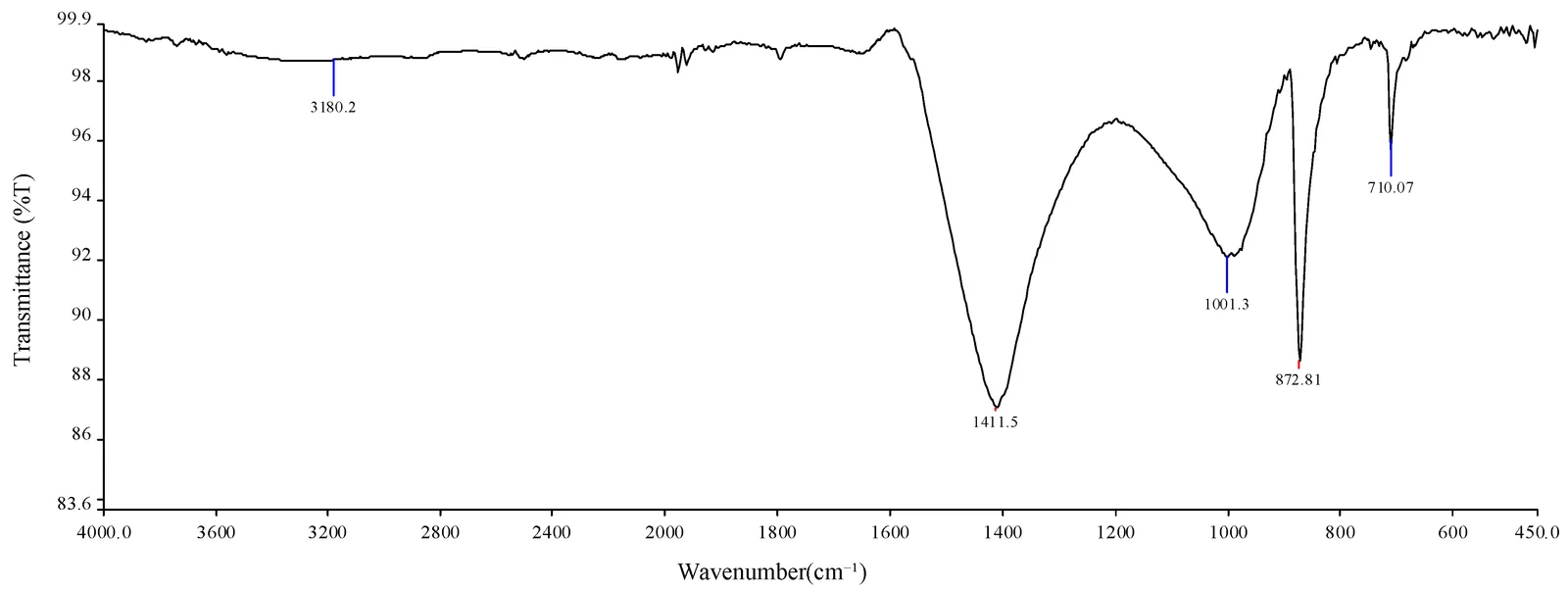
FTIR spectrum of the representative FA50–MK50–SS/SH2.5 mixture.

FTIR analysis was performed using a PerkinElmer 400 FTIR spectrometer (Spotlight 400 Imaging System, PerkinElmer Inc., Waltham, MA, USA). The spectrum of the representative FA50–MK50–SS/SH2.5 mixture is shown in Figure 12. The characteristic Si–O–T (T = Si or Al) stretching band near 1000 cm^−1^ is consistent with an alkali-activated aluminosilicate bonding environment and may reflect contributions from calcium-containing products. The band near 1420 cm^−1^ is associated with carbonate C–O vibrations, consistent with the carbonate-related XRD reflections. The band near 3180.2 cm^−1^ is associated with O–H stretching and is consistent with hydroxyl groups or retained or reabsorbed water; it is not assigned to the H–O–H bending mode. Low-intensity bands in the 450–800 cm^−1^ fingerprint region are associated with Si–O bending vibrations and residual silicate phases, including quartz and mullite.

### 4.3. Thermogravimetric Analysis (TGA/DTG)

**Figure 13:**
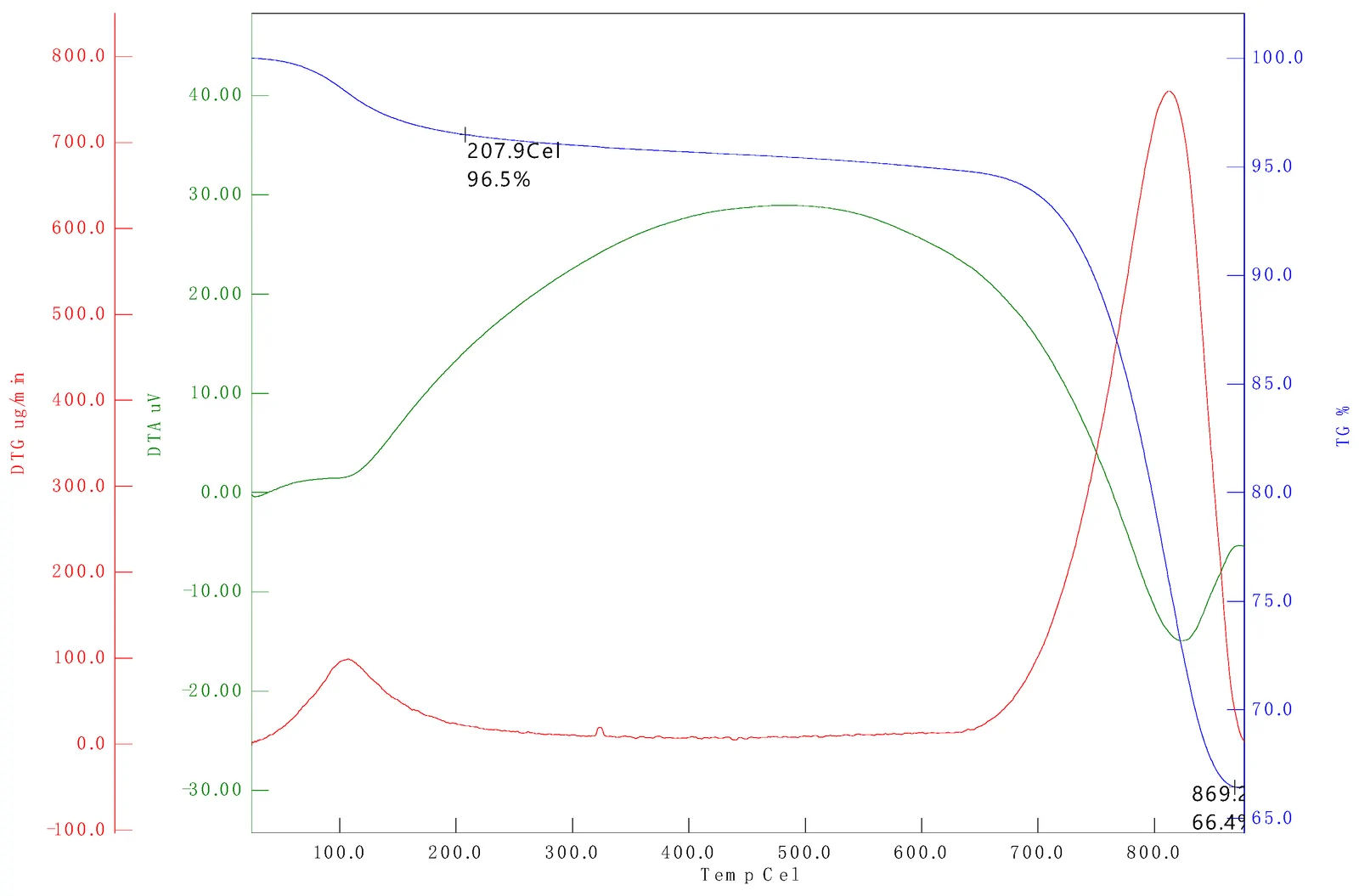
TGA/DTG curves of the representative FA50–MK50–SS/SH2.5 mixture after exposure to 300 °C.

Figure 13 presents the TGA/DTG curves of the representative FA50–MK50–SS/SH2.5 mixture, obtained using a Hitachi STA 7300 TG/DTA analyzer. Mass loss occurred in three principal temperature regions. The initial event at 25–200 °C, with a DTG peak near 110 °C, was attributed mainly to the evaporation of free and weakly bound water, consistent with the FTIR observations. Because the specimen had already been exposed to 300 °C, the free water released in this event most likely corresponds to moisture re-adsorbed after cooling rather than to water present before heating. Gradual mass loss between 200 and 600 °C was associated with dehydration and dehydroxylation of the reaction matrix, including Si–OH and Al–OH groups. Comparable dehydration- and dehydroxylation-related mass losses up to approximately 200–400 °C have been reported for ambient-cured MK-based geopolymer composites [57]. The higher-temperature event reported at 700–870 °C was consistent with carbonate decomposition and CO_2_ release. These carbonates may have formed through atmospheric carbonation, although contributions from carbonate-bearing raw materials or aggregates cannot be excluded.

### 4.4. FE-SEM Analysis

Field-emission scanning electron microscopy coupled with energy-dispersive X-ray spectroscopy (FE-SEM/EDX; ZEISS GeminiSEM 500, 10 kV, Carl Zeiss Microscopy GmbH, Oberkochen, Germany) was used to examine local morphology and elemental composition in the representative FA50–MK50–SS/SH2.5 mixture after heating. These observations provide qualitative context for the mechanical results; they do not measure bulk porosity, crack density, interfacial bond strength, or the extent of precursor reaction.

**Figure 14:**
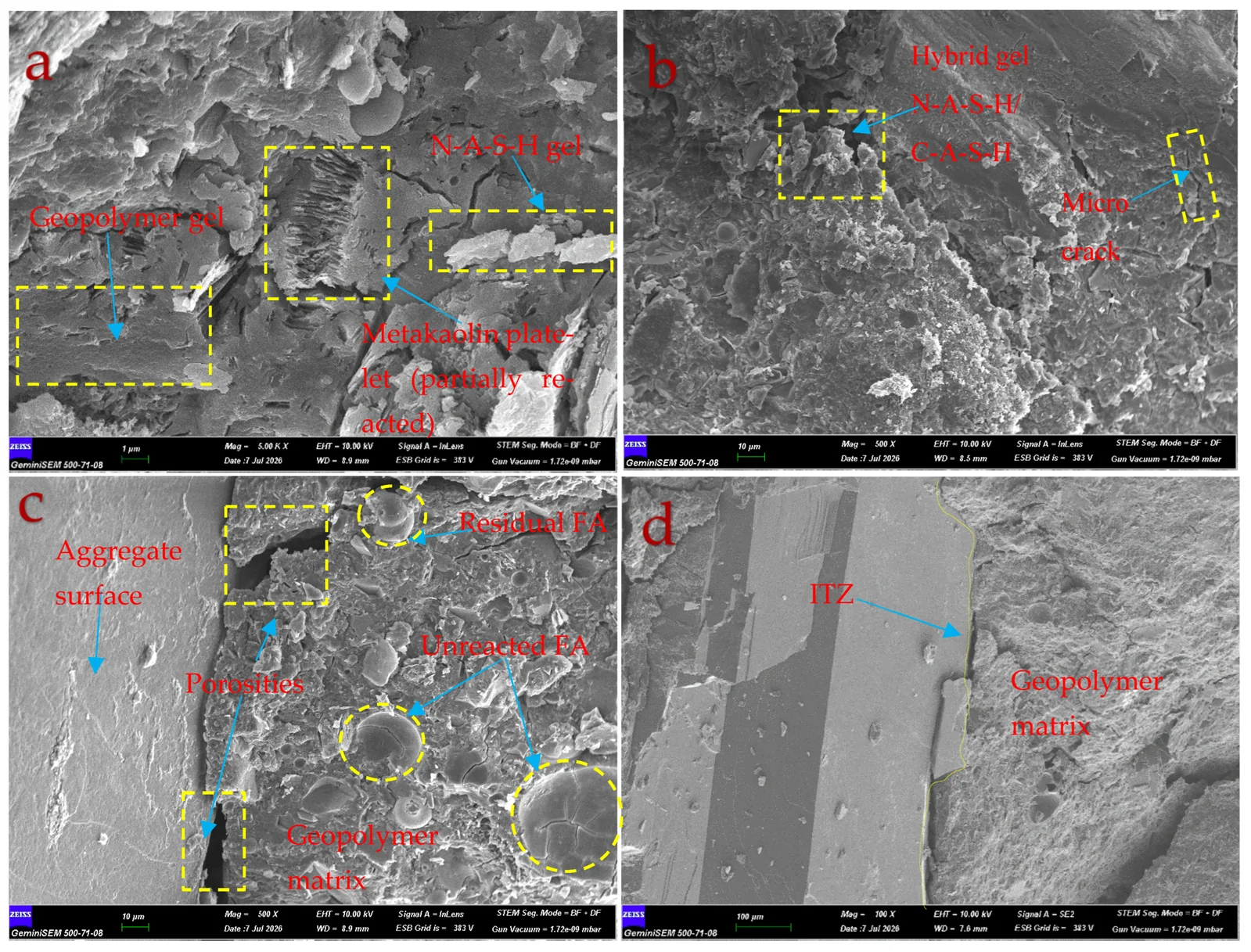
FE-SEM images of the representative FA50–MK50–SS/SH2.5 mixture after exposure to 300 °C: (**a**) matrix with a partially reacted metakaolin platelet; (**b**) matrix region with a microcrack; (**c**) aggregate surface, residual fly ash particles, and pores; (**d**) aggregate–matrix interfacial transition zone (ITZ). Labels referring to N-A-S-H and C-A-S-H gels are tentative interpretations based on morphology and local EDX composition, not phase-resolved identifications.

Figure 14 shows locally continuous matrix regions together with residual particles, voids, and microcracks. Plate-like and approximately rounded particles are morphologically consistent with residual MK- and FA-derived constituents, respectively, although morphology alone cannot establish particle identity or reaction extent. A well-embedded residual particle may contribute to packing and load transfer; a poorly bonded boundary can instead act as a stress concentrator or a crack-initiation site. The mechanical significance therefore depends on the continuity of the surrounding matrix and the quality of the local interface, rather than simply on the presence of unreacted material.

The matrix and aggregate interface in Figure 14 includes apparently continuous regions, but the absence of a visible crack in one field of view does not demonstrate uniformly strong adhesion. Conversely, observed cracks may interrupt load paths even when neighboring regions remain compact. Matrix contraction during water loss and thermal-strain mismatch around aggregates or residual particles offer plausible routes to such discontinuities. Compression can partly redistribute stress around local defects, whereas splitting failure is particularly sensitive to a connected weak path. These considerations connect the observed heterogeneity with residual mechanical capacity without assigning a quantitative damage contribution to individual features. The images were obtained from one composition after heating. Without paired pre-heating observations, thermal cracking cannot be separated conclusively from pre-existing shrinkage cracks or preparation artifacts. Likewise, visually compact regions do not establish a reduction in bulk pore connectivity. The SEM annotations referring to N-A-S-H or C-(A)-S-H are tentative compositional interpretations that require complementary phase-resolved characterization. The combined XRD, FTIR, and EDX evidence is consistent with an alkali-activated, calcium-containing aluminosilicate matrix, but cannot uniquely separate these gel phases. This interpretation also avoids attributing superior strength to the midpoint mixture, which was selected for representative characterization rather than maximum performance.

### 4.5. EDX Analysis

**Figure 15:**
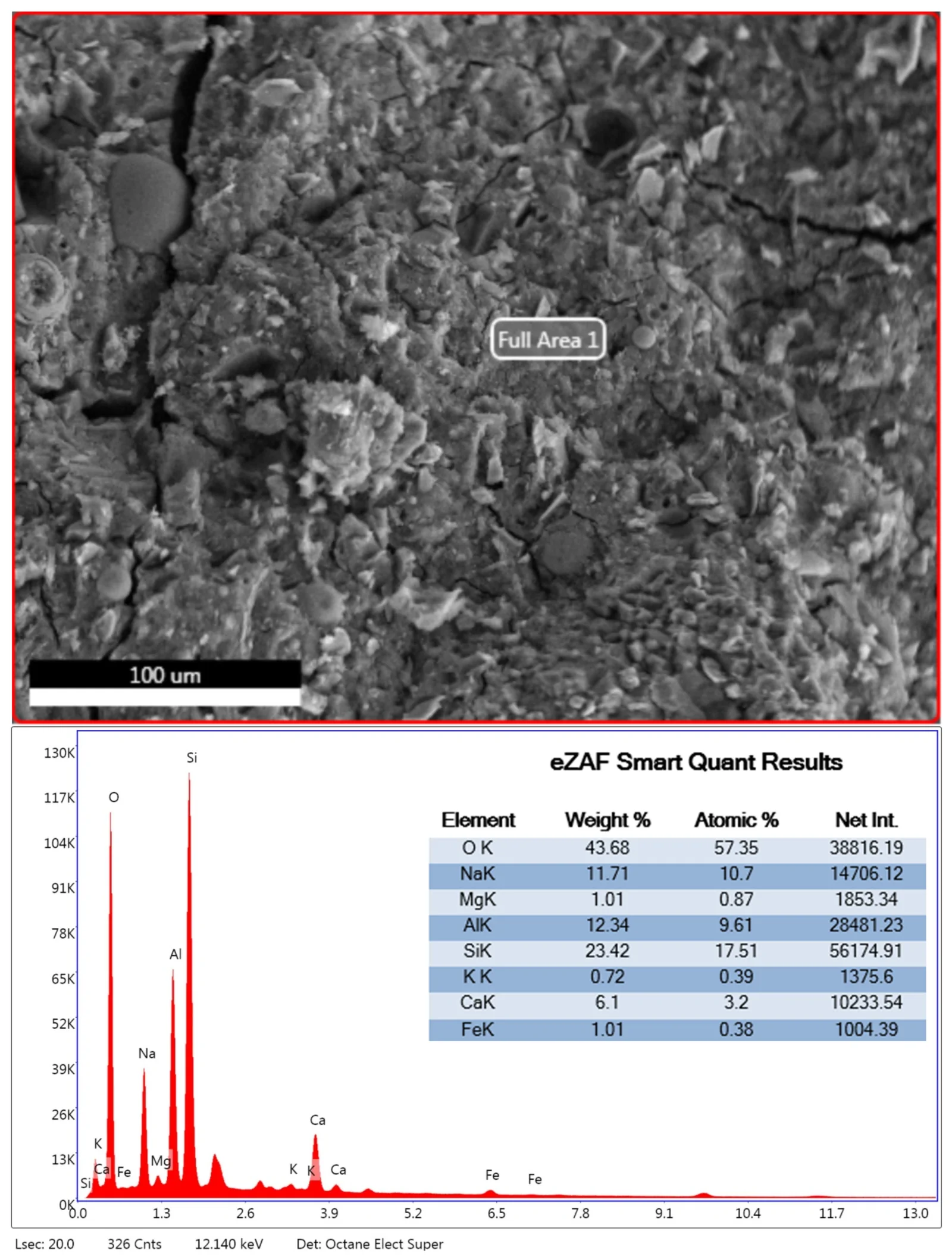
EDX analysis of the representative FA50–MK50–SS/SH2.5 mixture after exposure to 300 °C.

The EDX spectrum of the representative FA50–MK50–SS/SH2.5 mixture contained O (43.68 wt.%), Si (23.42 wt.%), Al (12.34 wt.%), Na (11.71 wt.%), and Ca (6.10 wt.%) (Figure 15). The combined Si–Al–Na composition is compatible with an alkali-activated aluminosilicate matrix, while measurable Ca is consistent with the contribution of slag-derived constituents. These local elemental proportions do not distinguish reacted gels from residual precursor or carbonate contributions, and do not uniquely identify N-A-S-H or C-(A)-S-H. Likewise, Na detection does not quantify the degree of dissolution, alkali incorporation, or chemical stability because both matrix-associated and residual alkali species may contribute. EDX therefore complements the morphological observations but does not independently demonstrate pore filling, reduced permeability, enhanced interface strength, or the mechanism of the measured strength loss.

## 5. Machine-Learning-Based Analysis, Optimization, and Validation Results

**Figure 16:**
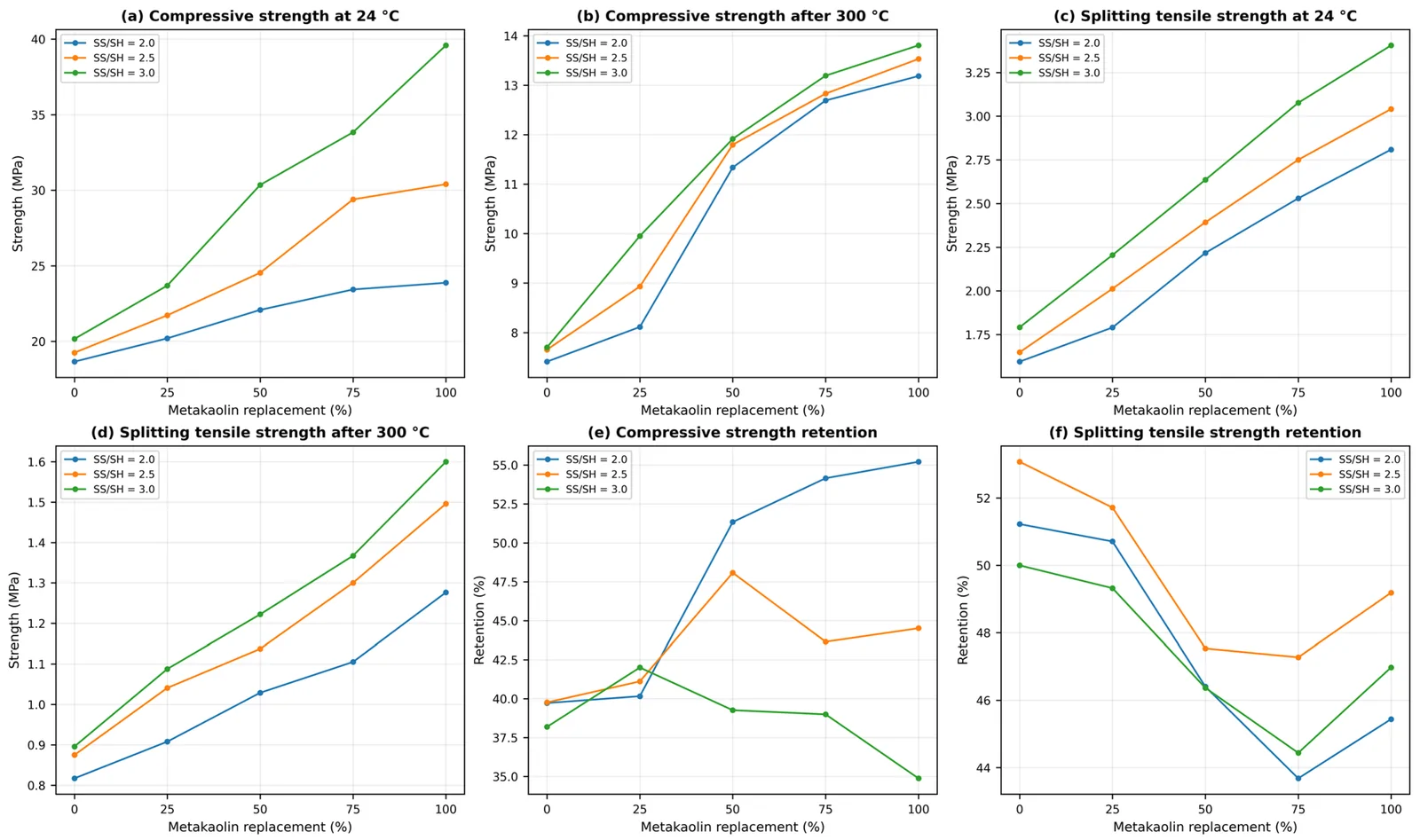
Interaction effects of MK replacement and SS/SH ratio: (**a**) compressive strength at 24 °C; (**b**) residual compressive strength after 300 °C; (**c**) splitting tensile strength at 24 °C; (**d**) residual splitting tensile strength after 300 °C; (**e**) compressive strength retention; (**f**) splitting tensile strength retention.

Mechanical responses showed a strong dependence on mixture composition: the dominant effect was the replacement of fly ash with metakaolin, while the SS/SH ratio had a weaker effect. As can be seen in Figure 16, the increase in MK content generally improved the four mechanical responses, and the highest CS_24_, CS_300_, STS_24_, and STS_300_ values were observed in MK100–SS/SH3.0 mixture, i.e., 39.588, 13.807, 3.405, and 1.600 MPa, respectively. The lowest responses were predominantly observed in FA-rich mixtures prepared at an SS/SH ratio of 2.0, suggesting that the replacement of fly ash with MK was the dominant factor controlling both the ambient-temperature strength and the residual strength after exposure to 300 °C.

The increase in ambient-temperature compressive strength was significantly influenced by MK content and the SS/SH ratio; at SS/SH = 2.0, CS24 increased only moderately (by 28%) from 18.660 MPa (at MK0) to 23.880 MPa (at MK100), whereas at SS/SH = 3.0, CS24 increased from 20.167 MPa to 39.588 MPa over the same MK range. Therefore, the improvement in strength with increased MK content was much greater when a higher SS/SH ratio was used. This difference explains the non-parallel trends in the CS24 panel in Figure 16 and indicates that the effect of MK should not be considered independently of the activator ratio. The increased availability of reactive aluminosilicate species provided by MK may explain this behavior, because a higher SS/SH ratio increases the soluble silicate content, which may facilitate geopolymerization and the formation of a more continuous reaction-gel network.

It is important to note, however, that this interpretation is a mechanistic hypothesis rather than a microstructurally verified conclusion, because the statistical analysis addresses macroscopic mechanical properties. Nonetheless, the strength–composition relationship provides clear statistical evidence of an interaction between MK replacement and activator composition for CS24. The post-heating compressive strength response presented a different picture. Like CS24, CS300 increased with MK content, but the differences between the three SS/SH curves became smaller at higher MK contents (Figure 16); for example, at MK100, CS300 ranged only from 13.186 to 13.807 MPa for SS/SH ratios of 2.0–3.0. This convergence implies that, after thermal exposure, the initial activator ratio became less influential and precursor composition remained the primary governing factor. A similar but weaker convergence was observed for splitting tensile strength, for which an activator-related difference persisted after heating (Section 3.2).

**Figure 17:**
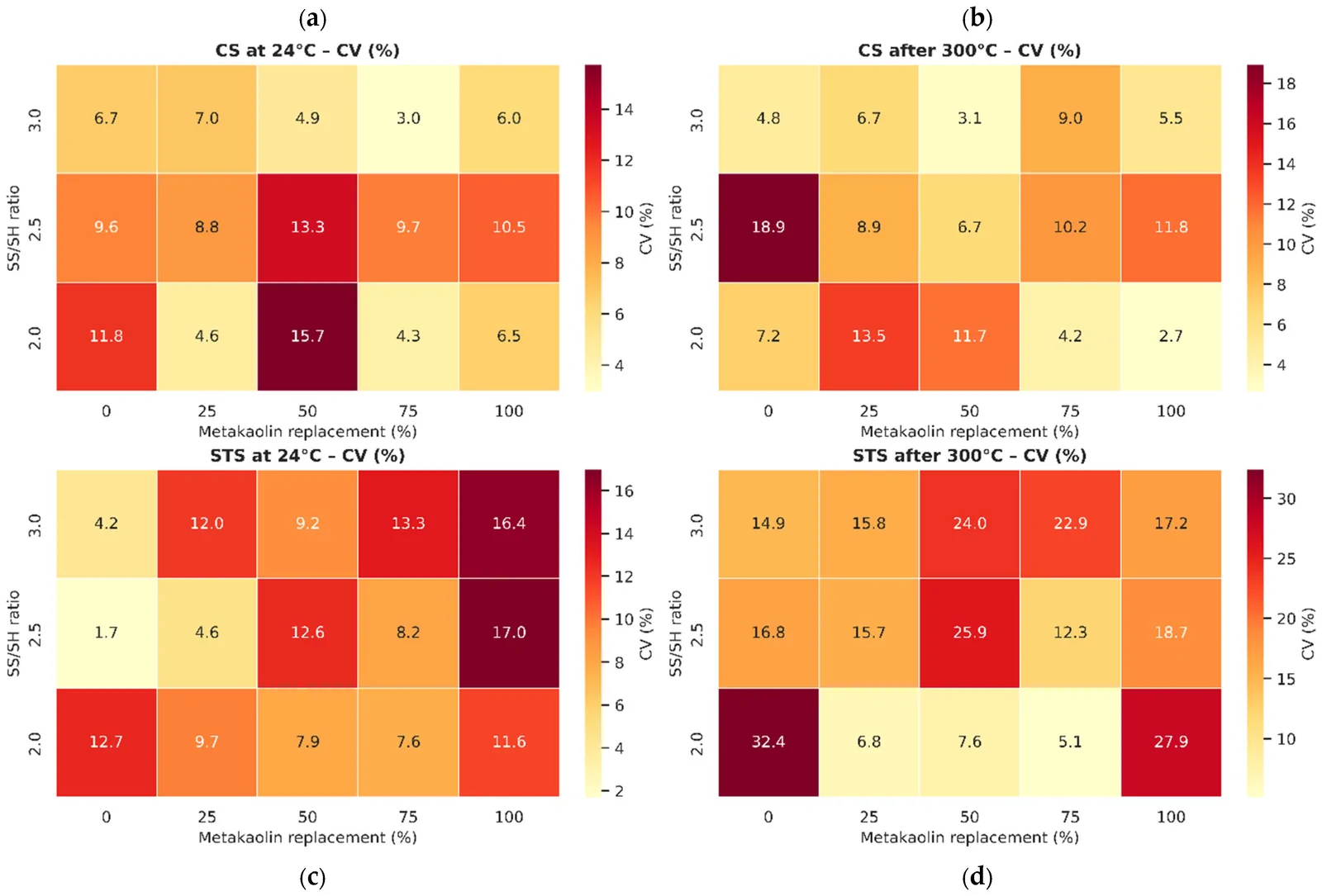
Experimental coefficients of variation (CV): (**a**) compressive strength at 24 °C; (**b**) residual com-pressive strength after 300 °C; (**c**) splitting tensile strength at 24 °C; (**d**) residual splitting tensile strength after 300 °C.

Both STS_24_ and STS_300_ increased progressively with MK content, and the mixtures with SS/SH = 3.0 produced the highest tensile strengths overall, as seen in Figure 16. The near-monotonic increase in tensile strength is consistent with improved matrix stress transfer; however, the splitting test does not independently quantify resistance to crack initiation or propagation. The largest specimen-level variability occurred in the post-heating tensile response, as shown by the CV maps in Figure 17: CVs ranged from approximately 3.0% to 15.7% for CS24, from 2.7% to 18.9% for CS300, and from 1.7% to 17.0% for STS24, whereas STS300 reached a maximum CV of 32.4%. Among them, MK0-SS/SH2.0 had the highest variability in STS300, followed by MK100-SS/SH2.0 and MK50-SS/SH2.5, with CVs of 32.4%, 27.9%, and 25.9%, respectively.

The greater scatter after heating is consistent with the sensitivity of splitting tensile strength to localized cracks and specimen-scale heterogeneity. Because the post-heating responses were measured using only two specimens per development mixture, the CV maps in Figure 17 provide imprecise estimates of mixture-specific variability. Model-based uncertainty assessment and three-specimen evaluation of selected compositions provide complementary evidence, but do not overcome the limited replication of the original development dataset.

**Figure 18:**
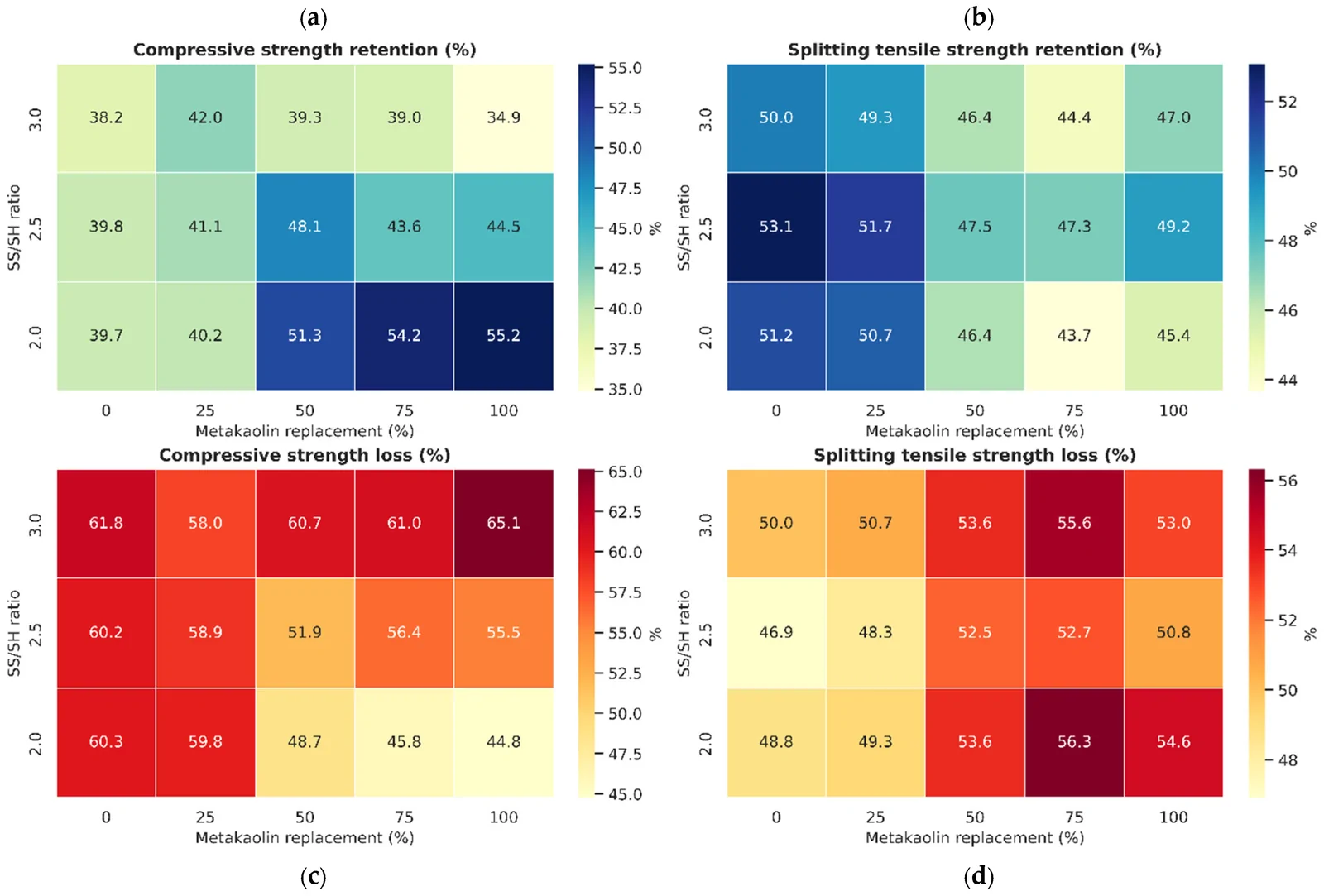
Thermal performance after exposure to 300 °C: (**a**) compressive strength retention; (**b**) splitting tensile strength retention; (**c**) compressive strength loss; (**d**) splitting tensile strength loss.

The thermal-retention maps in Figure 18 clearly show that the highest absolute strength under a given condition does not necessarily correspond to the highest proportional retention of strength. Compressive strength retention varied from 34.9% to 55.2%, and splitting tensile strength retention varied from 43.7% to 53.1%, indicating that exposure to 300 °C caused a larger proportional reduction in compressive strength than in splitting tensile strength in most mixtures. The highest compressive strength retention of 55.2% was obtained for MK100–SS/SH2.0. However, when the SS/SH ratio was increased to 3.0 for the same MK content, the compressive strength retention was only 34.9%, although this mixture had the highest absolute strength values both before and after heating. This is an important design trade-off: the higher the SS/SH ratio, the better the initial strength, but the smaller the proportion of that strength retained after heating, because retention also depends on the much higher ambient-strength denominator; it does not by itself demonstrate greater thermal damage.

In contrast, SS/SH = 2.0 resulted in reduced ambient-temperature strength but generally preserved a larger fraction of the original compressive capacity at high MK contents, as shown by the interaction plots in Figure 16. For SS/SH = 2.0, compressive strength retention increased markedly beyond MK25, reaching 51.3%, 54.2%, and 55.2% at MK50, MK75, and MK100, respectively; for SS/SH = 3.0, retention decreased from 42.0% at MK25 to 34.9% at MK100. Thus, the effect of MK on relative thermal performance was highly dependent on the activator ratio: MK enhanced retention at SS/SH = 2.0 but reduced it at SS/SH = 3.0. Lower strength retention observed in the high-strength mixtures could be associated with greater losses of physically and chemically bound water, thermal shrinkage incompatibility, or microcracking in a highly polymerized initial matrix, although these mechanisms were not quantified separately in this study.

However, this does not imply that SS/SH = 3.0 mixtures performed poorly after heating: they still achieved the highest absolute post-heating strengths but experienced a greater proportional loss because of their very high initial strengths at ambient temperature. Thus, absolute residual strength and normalized strength retention must be considered complementary, rather than interchangeable, performance indicators because they provide different insights into the material’s behavior. Retention of splitting tensile strength exhibited a clear dependence on mixture composition: the highest value (53.1%) was observed for MK0-SS/SH2.5, and the lowest (43.7%) for MK75-SS/SH2.0 (Figure 18). Increasing MK generally lowered STS retention from MK0 to MK75, with a partial recovery at MK100; this non-monotonic trend contrasts with the nearly monotonic increase observed in absolute STS300 and indicates that improvements in absolute tensile capacity do not directly translate into higher proportional thermal retention.

**Figure 19:**
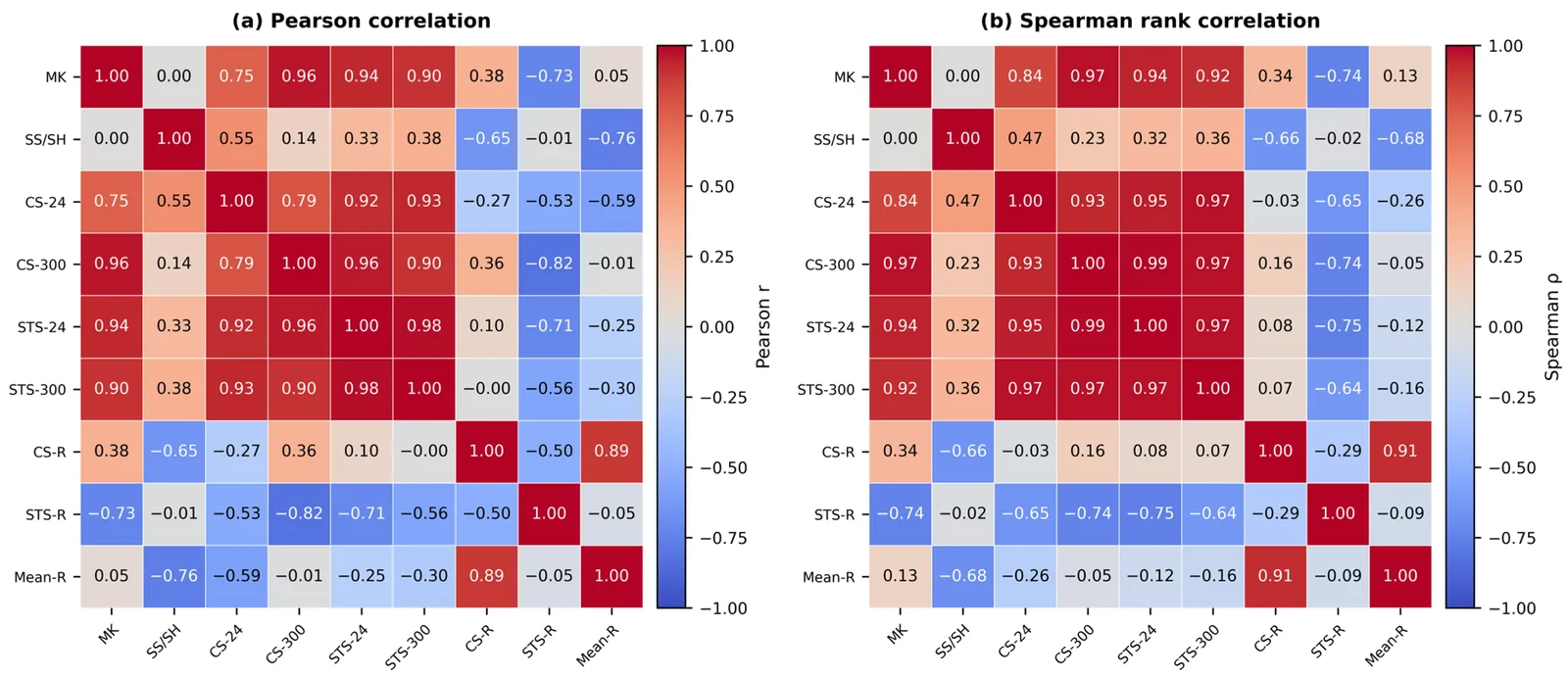
Correlation matrices for mixture parameters, mechanical responses, and thermal-retention indi-cators: (**a**) Pearson correlation; (**b**) Spearman rank correlation.

These observations are further supported by the correlation matrices in Figure 19, which show that MK was positively correlated with all four absolute strength responses. The Pearson coefficients were 0.75, 0.96, 0.94, and 0.90 for CS_24_, CS_300_, STS_24_, and STS_300_, respectively, and the corresponding Spearman coefficients were 0.84, 0.97, 0.94, and 0.92. The strong agreement between the Pearson and Spearman coefficients indicates that these correlations were not dominated by a few extreme mixtures but were largely monotonic across the examined design space. Consequently, the strongest correlation with MK was found for CS300, suggesting that the precursor composition is particularly important for the residual compressive strength after thermal exposure. On the other hand, MK was negatively correlated with the retention of splitting tensile strength, with Pearson and Spearman coefficients of −0.73 and −0.74, respectively, indicating that MK replacement tends to increase the absolute tensile strength while decreasing the percentage of tensile strength retained after heating.

These seemingly conflicting trends are expected because retention is a ratio-based response that depends on concurrent changes in ambient-temperature and post-heating strength. The SS/SH ratio was moderately correlated with CS24 (r = 0.55) but only weakly with CS_300_, STS_24_, and STS_300_. Conversely, SS/SH was negatively correlated with compressive-strength retention and the mean retention index, with Pearson coefficients of −0.65 and −0.76, and Spearman coefficients of −0.66 and −0.68 for those two measures, respectively (Figure 19). These correlations support the observation that increasing SS/SH tends to improve ambient-temperature strength more consistently than it improves proportional residual performance. Strong positive correlations were also observed among the four absolute mechanical responses, including STS24 and STS300 (Pearson coefficient = 0.98; Spearman coefficient = 0.97); CS300 was also strongly correlated with STS24 (Pearson coefficient = 0.96; Spearman coefficient = 0.99).

Overall, these correlations suggest that mixture modifications that enhance geopolymer matrix development generally improve multiple mechanical responses simultaneously; however, the weaker (and sometimes negative) correlations between absolute strength and retention metrics indicate that thermal retention is a distinct design objective. Hence, a mixture selected to maximize ambient-temperature strength will not necessarily be the same as a mixture selected for robust post-heating performance. This result provides the main motivation for treating strength and thermal retention as separate objectives in the multi-objective optimization that follows.

**Figure 20:**
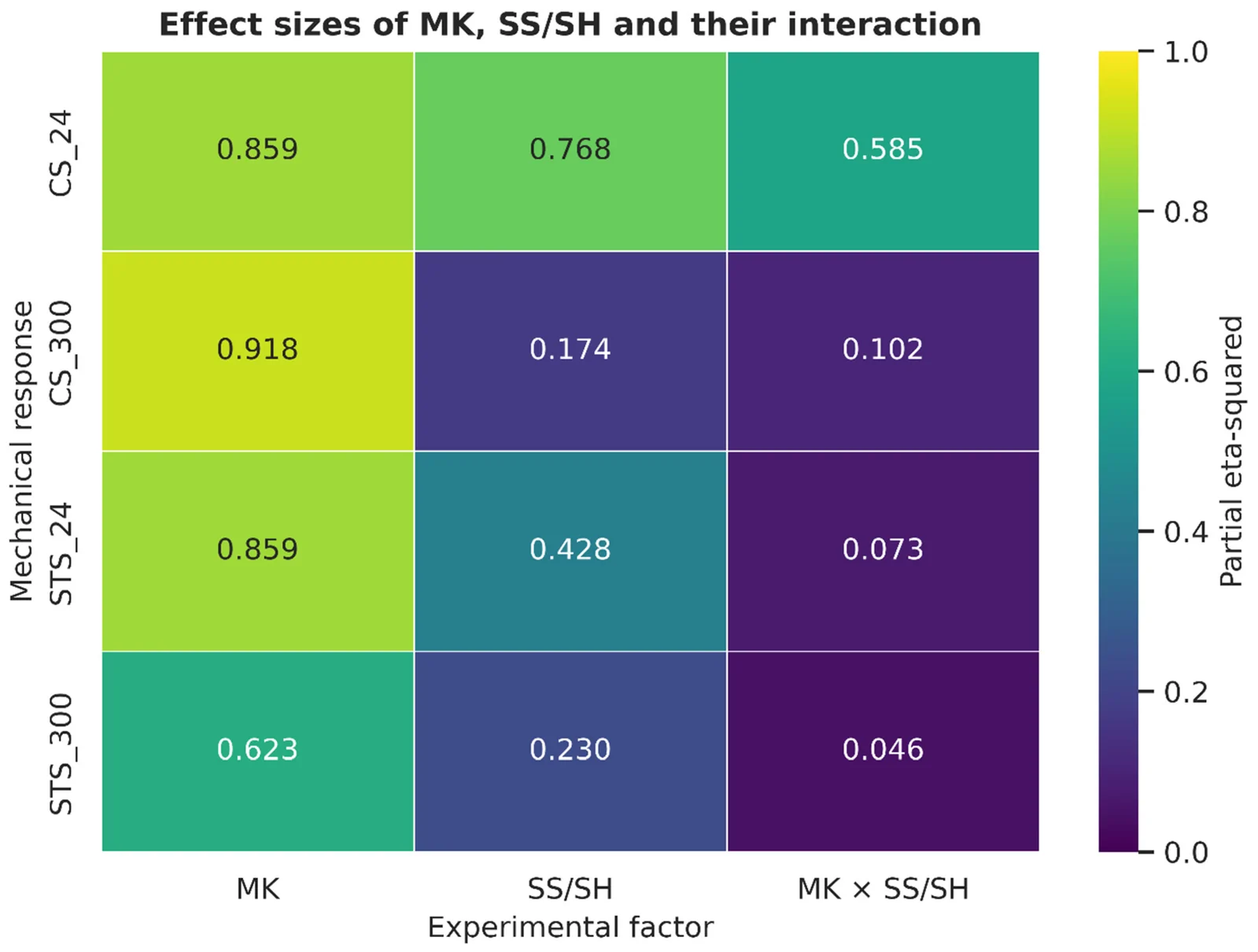
Partial eta-squared effect sizes of MK, SS/SH, and their interaction for the four mechanical responses.

Factorial analysis also confirmed that MK was the most important experimental factor for all four mechanical responses, with partial eta-squared values of 0.859 (CS_24_), 0.918 (CS_300_), 0.859 (STS_24_), and 0.623 (STS_300_), as shown in Figure 20, and all MK-related effects remained statistically significant after false-discovery-rate adjustment, meaning that the MK-dependent trends were not simply the result of pairwise associations, but instead reflected systematic differences across the full factorial design. The strongest MK effect was observed for CS300, with ηp^2^ = 0.918, consistent with the Pearson and Spearman correlation results presented in Figure 19, which further confirm that residual compressive strength was governed mainly by precursor composition. Although the effect of MK on STS_300_ was smaller than those observed for the other responses, its partial eta-squared value of 0.623 still represents a substantial contribution to post-heating tensile behavior. The SS/SH ratio had a large effect on CS_24_, with ηp^2^ = 0.768, and a moderate-to-large effect on STS_24_, with ηp^2^ = 0.428, as shown in Figure 20, and both effects remained statistically significant after FDR correction. In contrast, the effects of SS/SH on CS300 and STS300 were weaker, with partial eta-squared values of 0.174 and 0.230, respectively; however, these effects were not statistically significant after multiplicity adjustment, which is consistent with the convergence of the post-heating strength curves in Figure 16. The MK × SS/SH interaction was statistically significant only for CS24, with a partial eta-squared of 0.585, and was clearly reflected by the diverging CS24 curves in Figure 16. At low MK contents, varying SS/SH results in only minor differences in strength, while the impact of increasing SS/SH is far more substantial at MK75 and MK100; therefore, the ambient compressive-strength response cannot reasonably be approximated as the sum of two separate linear effects. For CS_300_, STS_24_, and STS_300_, the interaction effect sizes were 0.102, 0.073, and 0.046, respectively, and none of them remained significant after FDR adjustment, which is consistent with the relatively parallel response trends shown in Figure 16. Although minor local departures from parallel behavior were observed, they were insufficient to support strong factorial interaction effects for these responses.

**Figure 21:**
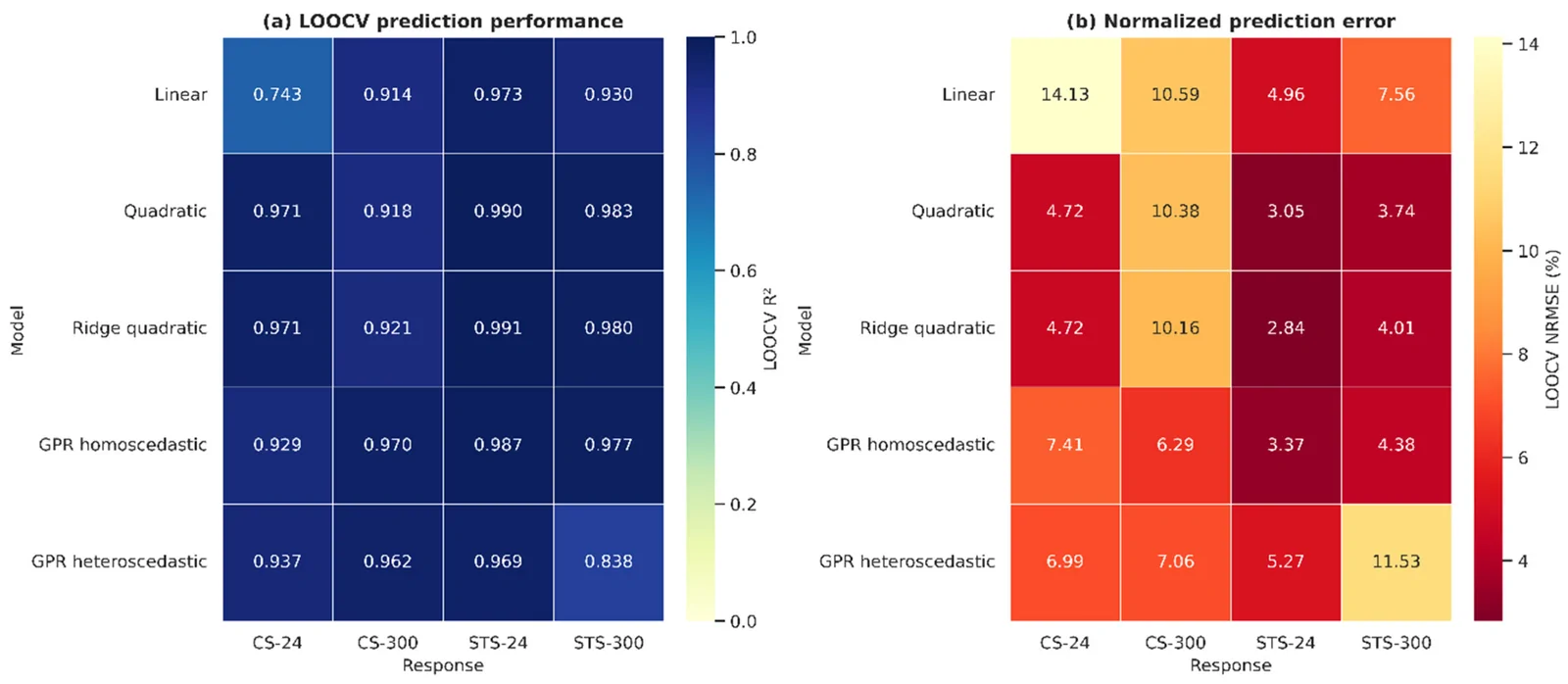
Baseline LOOCV comparison of linear, quadratic, ridge-quadratic, and GPR models: (**a**) coefficient of determination; (**b**) normalized prediction error.

Model comparison results revealed that a response-specific regression structure was more suitable than a general one; as shown in Figure 21, including quadratic terms led to a marked improvement in predictive performance over the linear baseline for all four mechanical responses. The largest improvement was observed for CS_24_, where the linear model yielded LOOCV R^2^ = 0.743 and NRMSE = 14.13%, while the quadratic and ridge-quadratic models yielded LOOCV R^2^ ≈ 0.971 and NRMSE = 4.72%, which was consistent with the strong MK × SS/SH interaction detected in the factorial analysis and the non-parallel ambient compressive-strength trends observed previously in Figure 16. This improvement supports the earlier conclusion that ambient compressive strength could not be adequately described by the independent additive effects of MK and SS/SH, and indicates that quadratic terms were required to capture both the curvature of the response and the stronger effect of SS/SH at higher MK replacement levels.

Ridge regularization offered only a minor numerical improvement over the unregularized quadratic model for CS_24_, but it did improve the stability of the polynomial coefficients in the face of the limited sample size; thus, the ridge-quadratic model was selected for CS_24_, which returned LOOCV R^2^ = 0.971, RMSE = 0.988 MPa, and NRMSE = 4.72%. Figure 21 shows the comparison between deterministic and probabilistic models. For CS24, GPR did not improve upon the quadratic alternatives: the heteroscedastic GPR model returned R^2^ = 0.937 and NRMSE = 6.99% while the homoscedastic GPR model returned R^2^ = 0.929 and NRMSE = 7.41%. This outcome can be attributed to the combined effects of strong interactions and spatially variable experimental uncertainty. With only 15 independent design points, the heteroscedastic model had to learn both a nonlinear response surface and mixture-specific noise, which increased the effective complexity of the modeling task; therefore, a reduced predictive accuracy of GPR for this response was expected.

For CS300, a different pattern was observed: the linear, quadratic, and ridge-quadratic models yielded LOOCV R^2^ = 0.914, 0.918, and 0.921, respectively, suggesting that polynomial expansion alone only provided limited improvement, while the homoscedastic Matérn GPR model improved R^2^ to 0.970 and NRMSE from 10.16% for ridge-quadratic regression to 6.29% (Figure 21). In terms of RMSE, this equates to a decrease from 0.650 MPa to 0.402 MPa, representing an improvement of 38.1%. The superior performance of GPR for CS300 indicates that the post-heating compressive response exhibited nonlinear variations that were not well captured by a fixed global quadratic function; unlike polynomial regression, GPR can adapt to local variations in response smoothness while maintaining a parsimonious structure through kernel-based regularization. The heteroscedastic GPR alternative also performed well for CS300 (R^2^ = 0.962; NRMSE = 7.06%), but it did not outperform the homoscedastic formulation, implying that the additional predictive value of including mixture-specific uncertainty did not justify the additional complexity of modelling the noise for this response.

Splitting tensile strength at 24 °C was modeled accurately by all model families, and even the linear model yielded R^2^ = 0.973, reflecting the largely monotonic response trend observed with increasing MK replacement, while the ridge-quadratic model was the best overall, yielding R^2^ = 0.991, RMSE = 0.051 MPa, and NRMSE = 2.84%. The relatively small difference between the quadratic and ridge-quadratic models indicates that the response is only slightly nonlinear, and regularization primarily serves to improve the stability of the polynomial coefficients rather than to correct significant overfitting. For STS300, the unregularized quadratic model yielded the best overall performance, with R^2^ = 0.983, RMSE = 0.029 MPa, and NRMSE = 3.74%. Ridge regularization slightly reduced R^2^ to 0.980 and increased NRMSE to 4.01%, suggesting that shrinkage was unnecessary for this relatively smooth response. The homoscedastic GPR model remained competitive, with R^2^ = 0.977, whereas the heteroscedastic formulation dropped to R^2^ = 0.838 and had NRMSE = 11.53%.

**Figure 22:**
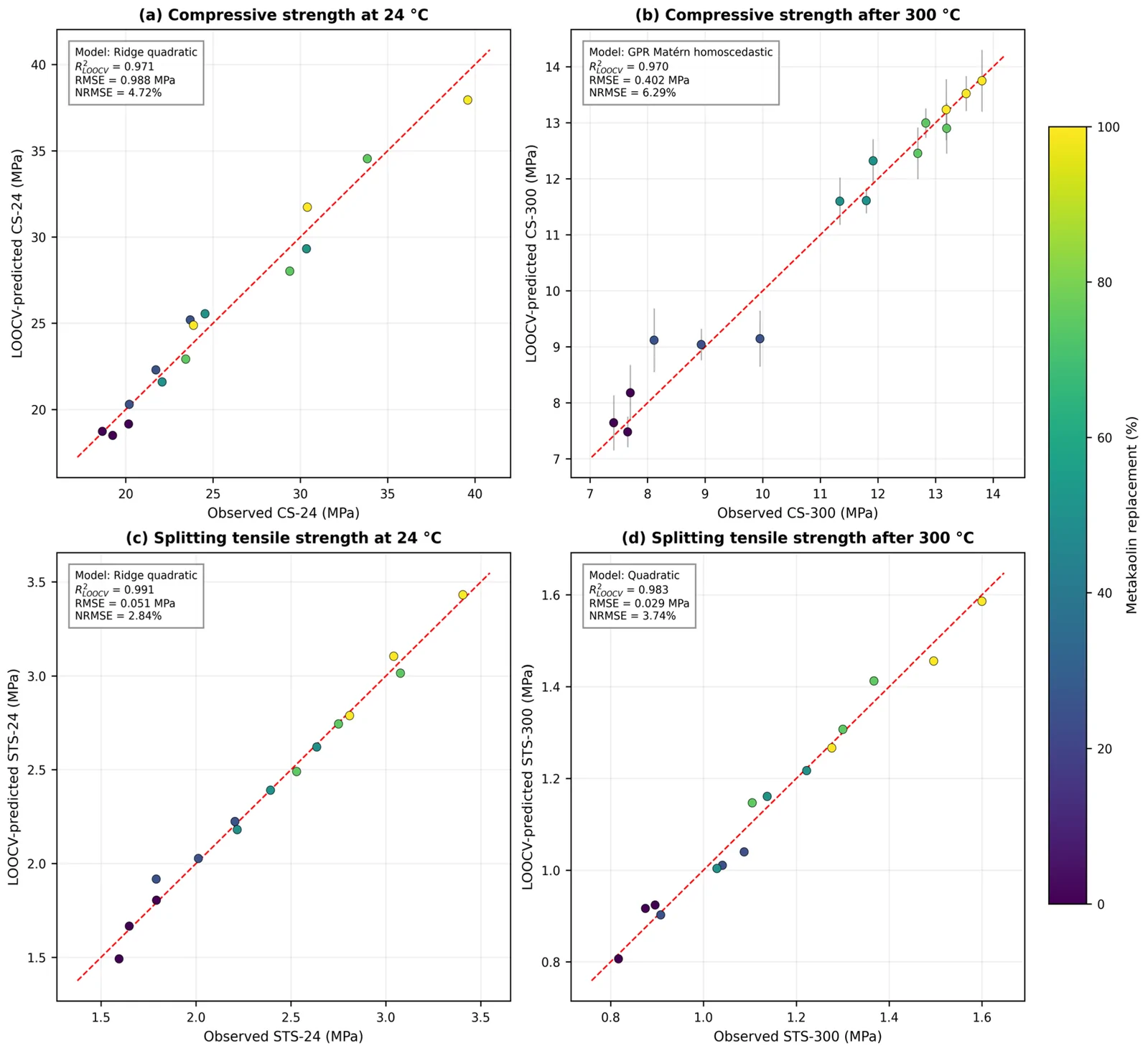
Out-of-fold parity plots: (**a**) compressive strength at 24 °C; (**b**) residual compressive strength after 300 °C; (**c**) splitting tensile strength at 24 °C; (**d**) residual splitting tensile strength after 300 °C. Colors indicate MK replacement. The red dashed lines indicate ideal agreement (predicted = observed); vertical error bars in (**b**) indicate 95% GPR prediction intervals.

However, because most post-heating experimental uncertainties were estimated from only two replicates, the mixture-specific variance estimates were inherently unstable. Therefore, treating these estimates as exact heteroscedastic noise inputs may have degraded, rather than enhanced, the generalization performance. The final out-of-fold parity plots in Figure 22 provide a more direct evaluation of model behavior than aggregate metrics alone. Each point in this figure represents a prediction made when the corresponding mixture was withheld from model fitting; the clustering therefore reflects leave-one-design-out performance within the original 15-point experimental domain and should not be interpreted as independent external evidence of broad generalization. For CS24, the ridge-quadratic predictions tracked the experimental trend across the full strength range, although some deviations were observed at the upper end of the response. In particular, the highest-strength mixture was moderately underpredicted, which is consistent with fitting a regularized global polynomial to a response that becomes increasingly steep at high MK and SS/SH levels. However, the absence of a systematic shift from the 1:1 line and the near-zero mean bias indicate that the model did not consistently over- or underpredict CS24.

The CS300 parity plot (Figure 22) shows the value of the GPR model, as the predicted values follow the 1:1 line, especially in the medium and high residual-strength range. The vertical error bars represent the uncertainty estimated by the GPR model, providing information in addition to point predictions. The LOOCV 95% prediction-interval coverage was 80%, indicating that the model captured most, but not all, of the held-out observations within the nominal prediction intervals. Nevertheless, interval coverage should be considered in conjunction with RMSE and MAE, and not as a stand-alone calibration metric, given the limited number of independent mixtures. The STS_24_ predictions were highly clustered around the 1:1 line across almost the entire response range, which is consistent with the highest LOOCV R^2^ and the lowest normalized error of the four responses, and the quadratic STS_300_ predictions also agreed with the experimental values with only minor localized deviations. Additionally, coloring the parity points according to MK content suggests that prediction errors were not systematically concentrated within any specific replacement range.

**Figure 23:**
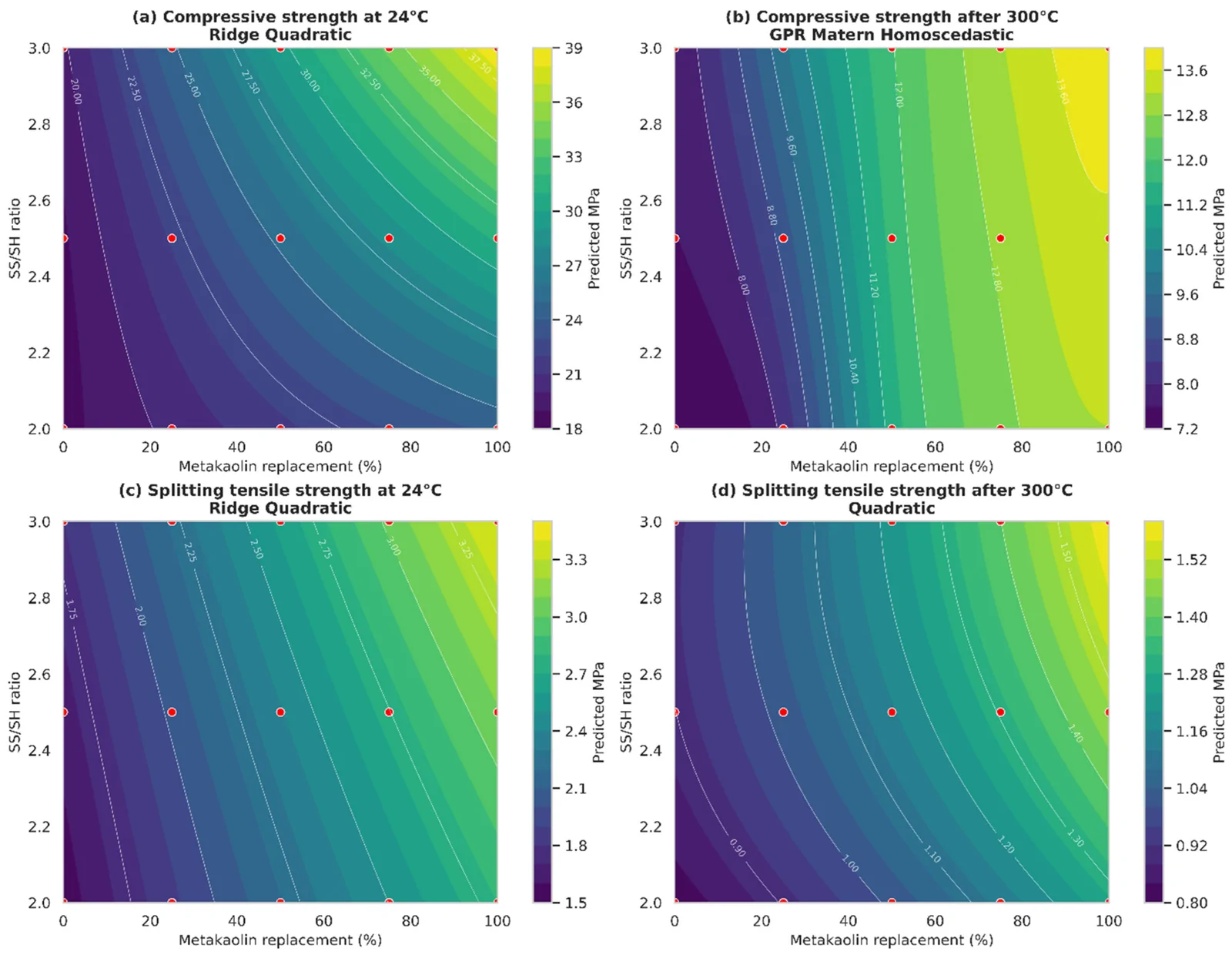
Predicted mechanical-response surfaces: (**a**) compressive strength at 24 °C; (**b**) residual compressive strength after 300 °C; (**c**) splitting tensile strength at 24 °C; (**d**) residual splitting tensile strength after 300 °C. Red points indicate the 15 experimental mixture compositions.

The response surfaces shown in Figure 23 translate the best-fit models into continuous composition–performance maps over the experimentally examined region, and the red points represent the 15 experimentally measured mixtures, so the colored regions should be considered as interpolation between experimentally substantiated design points, not unlimited extrapolation. The CS24 response surface showed the strongest two-variable curvature among the four responses (Figure 23a). At low MK contents, varying SS/SH produced little change in strength; however, as the level of MK replacement increased, the contours became increasingly curved and the strength gradient toward higher SS/SH ratios steepened considerably. This trend is consistent with the statistically significant MK × SS/SH interaction identified in Figure 20; the highest predicted ambient compressive strength was found close to the MK-rich boundary at SS/SH = 3.0, whereas the FA-rich region at low SS/SH produced the lowest predicted ambient compressive strength. The ridge-quadratic model exhibited a smooth and physically meaningful transition across the CS24 design space without negative or discontinuous predictions; however, the steep increase near the MK100–SS/SH3.0 corner indicates that the predictions in this boundary region are influenced to a certain extent by the high-strength endpoint; therefore, additional experimental mixtures between SS/SH = 2.5 and 3.0 will help to fine-tune the model response in this area.

The CS_300_ response surface was dominated primarily by the MK direction, as seen by the mostly vertical contours in Figure 23b, and residual compressive strength rose rapidly from MK0 up to about MK50 before approaching a flatter region at higher MK contents, in contrast, the variation along the SS/SH axis was limited. This surface geometry is consistent with the factorial analysis in which MK exhibited a very large effect size, while SS/SH and the MK × SS/SH interaction were not statistically significant after FDR correction. The anisotropic GPR kernel confirms this interpretation, since a shorter GPR length scale implies more rapid variation in the response. The fitted CS300 kernel assigned distinct characteristic length scales to the two input variables: a shorter characteristic length scale for MK than for SS/SH, confirming that CS300 was considerably more sensitive to MK replacement than to the activator ratio.

The STS_24_ surface showed a smooth and nearly monotonic increase with MK, together with a smaller but consistent increase with SS/SH, as seen in Figure 23c, and the contour lines were less curved than those of CS24, which agrees with the weak and statistically non-significant MK × SS/SH interaction. The effect of SS/SH was smaller than that of MK but still apparent across the design space, especially for MK-rich mixtures, and the STS_300_ surface also increased with MK and displayed only minor curvature in both input directions, as seen in Figure 23d. The highest predicted post-heating splitting tensile strength was observed near MK100–SS/SH3.0, while the lowest response occurred near the MK0–SS/SH2.0 corner. The surface smoothness justifies the choice of the unregularized quadratic model and explains why the more flexible GPR model did not improve LOOCV performance for this response.

**Figure 24:**
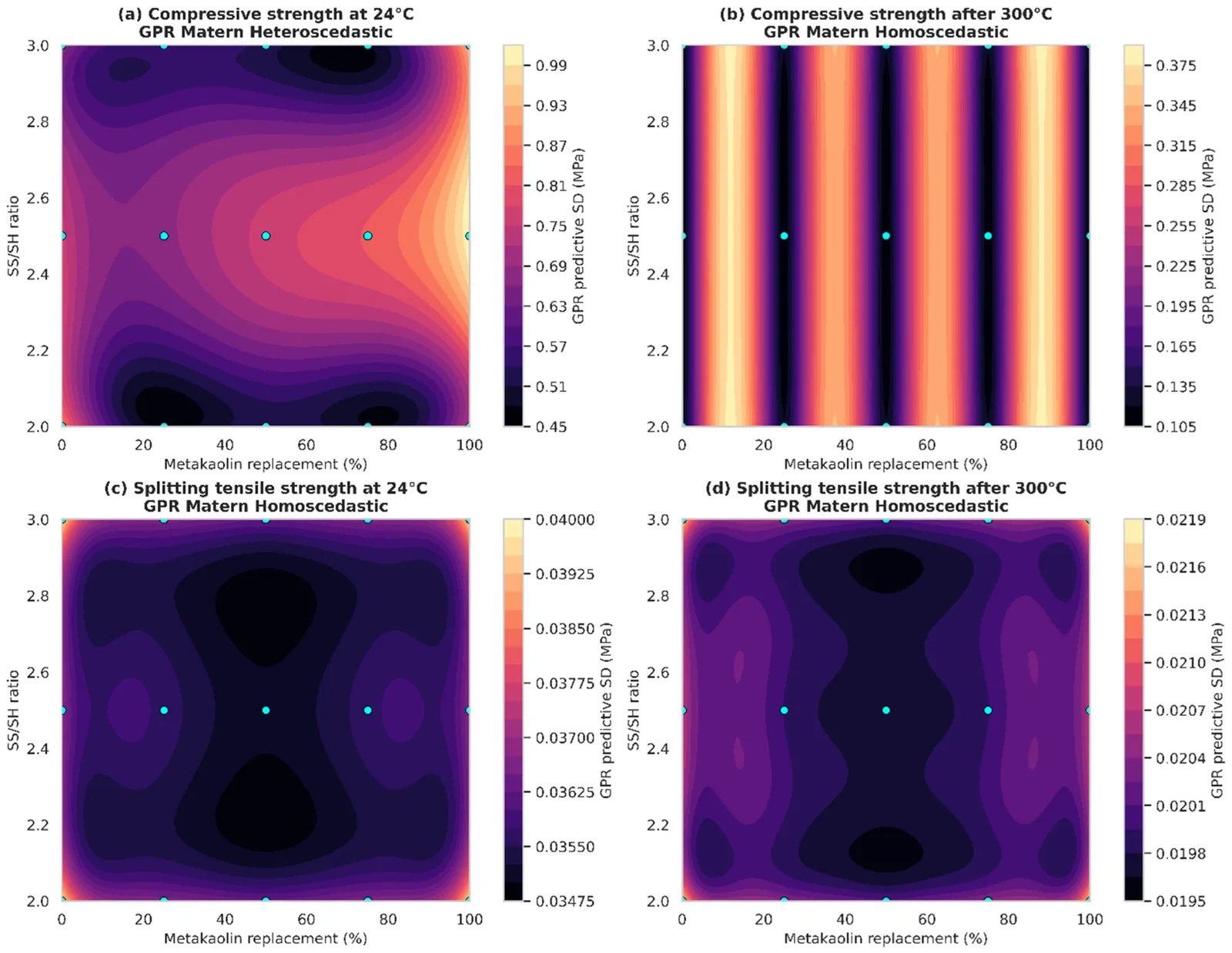
GPR predictive-standard-deviation maps: (**a**) compressive strength at 24 °C; (**b**) residual compressive strength after 300 °C; (**c**) splitting tensile strength at 24 °C; (**d**) residual splitting tensile strength after 300 °C. Blue points indicate the 15 experimental mixture compositions.

The GPR uncertainty maps in Figure 24 provide further insight into the reliability of model predictions throughout the design space; however, they should not be interpreted as strength-response surfaces. Instead, they show the model-estimated standard deviation of each predicted response, with lower values indicating greater model confidence and higher values indicating regions with increased prediction uncertainty. The heteroscedastic CS24 GPR model produced the largest absolute uncertainty among the four responses, with predictive standard deviations of approximately 0.45–1.00 MPa, as seen in Figure 24a, and the irregular uncertainty pattern is a result of both the nonlinear nature of the CS_24_ response, and the inclusion of composition-specific experimental noise. Higher uncertainty is observed near parts of the design boundary and in regions associated with greater experimental variability, which explains why heteroscedastic GPR was useful for robust uncertainty propagation, despite not outperforming ridge-quadratic regression in point-prediction accuracy.

The CS_300_ uncertainty map is characterized by clear vertical banding, as seen in Figure 24b, and uncertainty was lowest around the experimentally sampled MK levels and grew with distance between adjacent levels, with little variation along the SS/SH direction, which is a consequence of the anisotropic Matérn kernel, and further supports that the GPR model treated MK as the dominant direction of response variation. Predictive standard deviation stayed around 0.105–0.38 MPa, which was much lower than the CS_24_ uncertainty on an absolute scale, and the STS24 and STS_300_ uncertainty maps were more uniform, as seen in Figure 24c,d. Predictive standard deviation was around 0.035–0.040 MPa for STS24 and 0.0195–0.0219 MPa for STS_300,_ which indicates that the smooth tensile-strength trends were learned consistently throughout most of the investigated design domain, and the slight increase near boundaries and between experimental design levels remained, as expected for interpolation-based probabilistic models.

These differences in absolute uncertainty should be considered in the context of their respective response scales; for example, a predictive standard deviation of 0.8 MPa may be moderate for compressive strength but substantial for splitting tensile strength. Thus, the subsequent robust optimization used response-specific normalization and relative uncertainty rather than directly comparing raw standard deviations.

**Figure 25:**
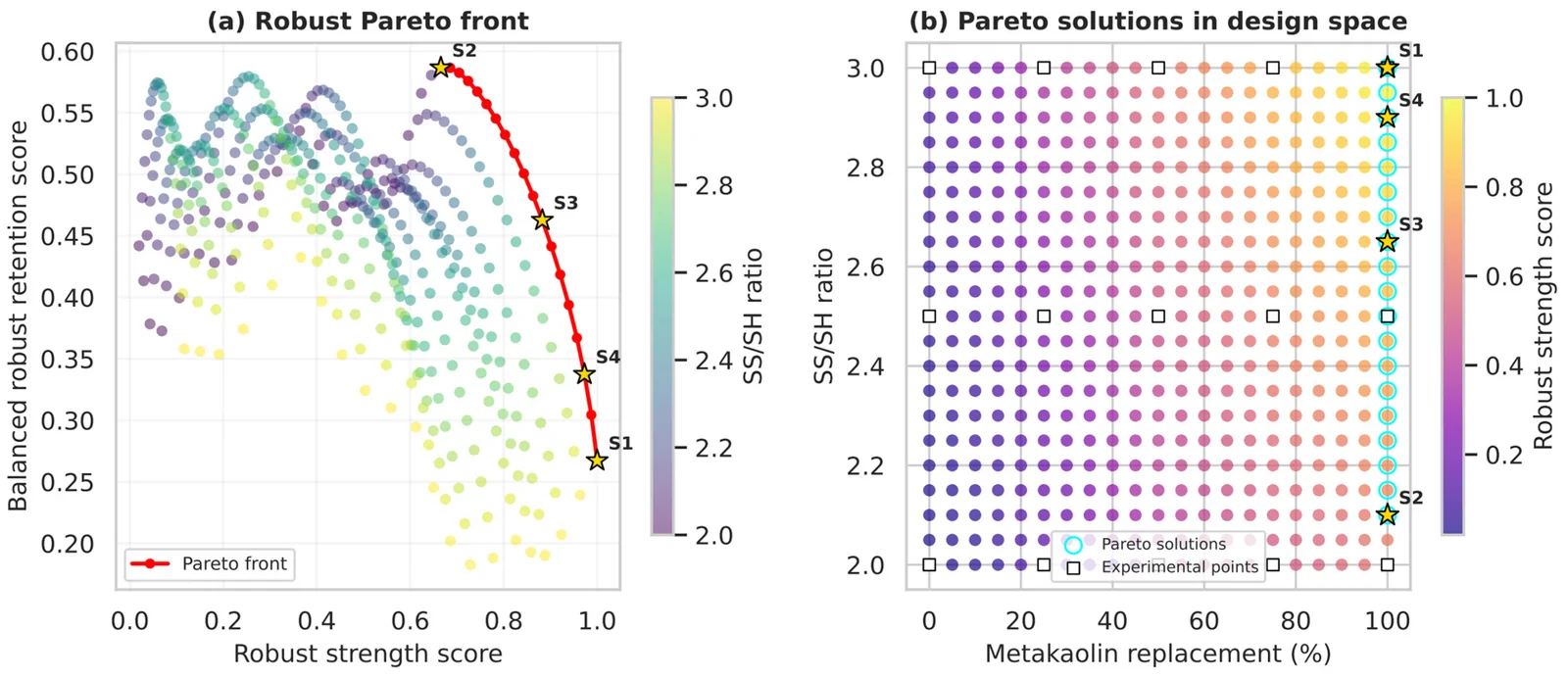
Uncertainty-aware multi-objective optimization: (**a**) robust Pareto front; (**b**) Pareto solutions in the design space. Stars identify selected candidates S1–S4.

The external-validation mixtures were selected directly from the uncertainty-aware optimization results shown in Figure 25, which display an apparent trade-off between lower-bound mechanical strength and balanced thermal-retention performance, as reflected in the robust Pareto front in Figure 25a. S2 was the solution with the maximum robust-retention score; S1 had the maximum robust-strength score; S3 was chosen as the main Pareto knee-point solution because it represents a balanced compromise between the competing objectives; and S4 was chosen as a reliable, high-strength solution located near the strength-dominant region of the Pareto front. The distribution of the optimized mixtures in the design space, as shown in Figure 25b, also demonstrated that all Pareto-optimal candidates converged on 100% MK replacement and that the SS/SH ratio determined their position along the strength–retention trade-off. The selected SS/SH ratios were 2.10, 2.65, 2.90, and 3.00 for S2, S3, S4, and S1, respectively. To determine whether these computationally predicted trade-offs can be reproduced experimentally, the four selected Pareto-optimal mixtures were tested alongside the R1 reference mixture, which contained 50% MK and had an SS/SH ratio of 2.50. S2, S3, and S4 were new compositions, whereas S1 (MK100–SS/SH3.0) and R1 (MK50–SS/SH2.5) were independent reproductions of design points from the original factorial program. For each validation mixture, four mechanical responses were measured on three independently prepared specimens, resulting in a total of 60 individual measurements and 20 response-design validation records. All validation data were kept fully independent and were not used for model fitting, hyperparameter selection, uncertainty estimation, or Pareto optimization.

The Pareto front should be read as a conditional strength–retention trade-off. Moving from the retention-oriented S2 candidate (SS/SH = 2.10) toward the strength-oriented S1 candidate (SS/SH = 3.00) favors the lower-bound strength score while sacrificing balanced proportional retention. At 100% MK, the experimental endpoint comparison illustrates why: ambient compressive strength changes substantially with SS/SH, while the residual compressive strengths remain relatively close. The retention advantage of the lower-ratio mixture therefore partly reflects its lower initial strength. Selection for service after heating must consider the absolute residual compressive and tensile capacities as well as retention; a high percentage alone does not establish adequate capacity.

All Pareto candidates lie at 100% replacement of the 400 kg/m^3^ FA–MK fraction. This boundary corresponds to 400 kg/m^3^ MK plus the fixed 125 kg/m^3^ GGBFS, rather than a binder consisting entirely of MK. Its selection means that the modeled mechanical objectives favored the MK-rich boundary of the investigated domain. It does not establish a universal optimum or prove that lower-MK alternatives are impractical. The observed reduction in slump with MK and SS/SH is relevant because placing and compaction can constrain a strength-favorable formulation. This constraint is likely to be most relevant at the MK-rich boundary, because higher MK contents and Na_2_SiO_3_/NaOH ratios increase paste and activator viscosity [47,48]. However, slump was not modeled quantitatively or imposed as a feasibility constraint, so the candidate set does not establish an optimum for workability.

S3 at SS/SH = 2.65 is therefore the distance-to-ideal compromise under the specified objectives, normalization, equal response weights, and GPR uncertainty estimates. Changing objective weights or introducing minimum slump and residual-strength requirements could change both the feasible set and the preferred mixture. Its experimental mechanical evaluation supports local prediction at that composition; it does not validate placement behavior, application-specific capacity, or overall fire resistance. Practical selection should screen candidate mixtures against application-specific fresh-state and residual-strength requirements, while recognizing that additional water or admixture changes would define a new formulation requiring verification. This joint treatment of strength, setting, and workability is consistent with the mix-design perspective of Hadi et al. [42].

Table 3 summarizes the predictive performance on the experimental evaluation set. Each response was evaluated using five mixture-level means: three newly tested compositions and two compositions independently reproduced from the original design. Descriptive R^2^ values ranged from 0.9062 to 0.9989, and MAPE ranged from 0.47% to 1.40%. These results indicate close agreement within this limited set; they do not establish broad model generalizability.

For the S3 knee-point mixture, the measured CS300 was 14.1867 MPa, compared with a predicted mean of 13.6173 MPa and an upper 95% prediction limit of 13.8362 MPa. The relative prediction error was 4.01%, indicating a local-interval miss despite a modest point-prediction error. Overall, 18 of 20 response means were within the original 95% prediction intervals, resulting in strict coverage of 90%. One borderline STS300 record was additionally covered after applying a ±0.005 MPa measurement-resolution tolerance, increasing adjusted coverage to 19 of 20 records (95%). This adjusted result serves as a supplementary diagnostic and does not demonstrate nominal 95% calibration for the original intervals.

## 6. Study Limitations and Future Studies

The proposed optimization should be viewed as a decision-support framework based on mechanical considerations, rather than as a complete mixture-design specification. The knee-point solution for 100% MK was obtained at SS/SH = 2.65; the Pareto-optimal ratios ranged from 2.10 (favoring retention of residual strength) to 3.00 (maximizing the conservative strength score). The composition range that would simultaneously satisfy the requirements for strength, residual performance, and workability could not be determined precisely because slump was not included as a quantitative ML response or as an optimization constraint. Future studies should incorporate replicate-level slump measurements, application-specific performance thresholds, and sensitivity analyses of scaled or shrunken experimental variance maps.

Thermal performance was evaluated at a single exposure temperature of 300 °C in a controlled laboratory environment. This moderate exposure provides insight into the initial effects of dehydration, shrinkage, and thermally induced microcracking, but does not replicate an actual fire, which can involve higher temperatures, non-uniform thermal gradients, sustained loading, and different heating and cooling regimes. The procedure did not include a standard time–temperature curve or fire-resistance criterion. It should not be considered a fire-resistance test. The results show the residual mechanical and microstructural properties after exposure to 300 °C, but they should not be directly extrapolated to more severe conditions. For MK-based geopolymer composites, a critical strength transition associated with partial melting and void formation has been reported at approximately 900 °C, and filler type and particle size strongly affected the residual strength at higher temperatures [57]. Only the representative FA50-MK50-SS/SH2.5 mixture was characterized physicochemically and microstructurally; therefore, compositional changes across the entire experimental domain could not be determined. Future work should investigate other mixtures, temperatures up to 600–800 °C, different exposure and cooling regimes, standardized fire curves, and thermomechanical loading.

The predictive results are also constrained by dataset size and replication structure. Model development was based on 15 mixture designs. CS300, STS24, and STS300 were each measured using two specimens at each design point. Thus, mixture-specific estimates of CV are limited in precision, and GPR-based uncertainty quantification cannot substitute for further experimental replication. The experimental evaluation consisted of five compositions per response: three newly evaluated compositions and two independent reproductions of existing design points. Thus, the resulting R^2^, error, and prediction-interval coverage metrics should be interpreted descriptively, rather than as evidence of broad generalizability. Further validation should involve additional compositions, replicates, material batches, and independent laboratory conditions.

## 7. Conclusions

The 15-mixture factorial study identified the following principal findings for ambient-cured fly ash–MK–slag geopolymer concrete:

- Increasing MK replacement and SS/SH reduced the slump and improved ambient strength. At SS/SH = 3.0, replacing 0% MK with 100% MK increased compressive strength from 20.17 MPa to 39.59 MPa and splitting tensile strength from 1.79 MPa to 3.41 MPa. MK was the dominant mechanical-performance factor; its interaction with SS/SH was statistically significant only for ambient compressive strength after multiplicity adjustment.
- Exposure to 300 °C reduced both strengths in all mixtures. Within the original factorial dataset, 100% MK at SS/SH = 3.0 produced the highest residual compressive and splitting tensile strengths of 13.81 MPa and 1.60 MPa, respectively. However, its compressive strength retention was 34.9%, compared with 55.2% at SS/SH = 2.0. Maximizing absolute strength, therefore, did not maximize proportional retention.
- For the representative FA50–MK50–SS/SH2.5 mixture, XRD and FTIR analyses were consistent with an alkali-activated aluminosilicate matrix, while TGA/DTG analyses indicated water removal, overlapping dehydration processes, and a higher-temperature event, consistent with carbonate decomposition. FE-SEM/EDX provided complementary, local evidence of residual particles, voids, microcracks, and aluminosilicate-bearing and calcium-bearing constituents. These qualitative observations neither uniquely identified specific gel phases nor quantified differences among mixtures.
- The response-specific models showed local predictive agreement for three newly tested and two independently reproduced compositions (MAPE: 0.47–1.40%). The original 95% prediction intervals contained 18 of the 20 response means, corresponding to 90% coverage. With only five evaluation means per response, these results provide preliminary evidence of local usefulness rather than broad generalizability or nominal 95% interval calibration.
- Uncertainty-aware optimization placed all Pareto candidates at 100% MK, with selected SS/SH ratios spanning 2.10–3.00 and a knee point at 2.65. This candidate balances conservative strength and thermal-retention objectives within the modeled domain; however, it is not a practical mixture specification because workability was not included as a quantitative constraint.
- The findings are limited to 15 development mixtures, limited replication, a single exposure temperature, and physicochemical characterization of a single representative composition. Further validation should incorporate workability constraints, additional compositions and material batches, and broader thermal exposure conditions before practical design recommendations are established.

## Figures and Tables

**Table 1:** Chemical composition of the precursors (wt.%).

Components	Fly Ash	GGBFS	Metakaolin
Al_2_O_3_	26.704	13.615	45.366
BaO	0.383	0.965	0
CaO	1.776	31.761	0.167
Cr_2_O_3_	0.023	0.013	0.018
CuO	0	0	0
Fe_2_O_3_	5.992	1.249	0.413
HfO_2_	0.001	0	0
K_2_O	3.371	0.789	0.056
MgO	2.192	5.325	0.072
Mn_3_O_4_	0.069	0.499	0
Na_2_O	0.009	0.058	0
NiO	0.02	0.009	0.012
P_2_O_5_	0.169	0.012	0.063
PbO	0	0	0
SiO_2_	54.279	41.394	52.148
SO_3_	0.16	1.424	0.073
TiO_2_	1.142	2.391	1.089
V_2_O_5_	0.023	0	0.03
ZnO	0.006	0	0
ZrO_2_	0.024	0.014	0.021
SrO	0.069	0.106	0.028

**Table 2:** Mixture proportions (kg/m^3^).

Sample	Fly Ash	Metakaolin	GGBFS	Na_2_SiO_3_	NaOH	SS/SH Ratio	Fine	Coarse
	(kg)	(kg)	(kg)	(kg)	(kg)		Aggregate (kg)	Aggregate (kg)
FA100%MK0%SS/SH3.0	400	0	125	393.75	131.25	3	500	1000
FA75%MK25%SS/SH3.0	300	100	125	393.75	131.25	3	500	1000
FA50%MK50%SS/SH3.0	200	200	125	393.75	131.25	3	500	1000
FA25%MK75%SS/SH3.0	100	300	125	393.75	131.25	3	500	1000
FA0%MK100%SS/SH3.0	0	400	125	393.75	131.25	3	500	1000
FA100%MK0%SS/SH2.5	400	0	125	375	150	2.5	500	1000
FA75%MK25%SS/SH2.5	300	100	125	375	150	2.5	500	1000
FA50%MK50%SS/SH2.5	200	200	125	375	150	2.5	500	1000
FA25%MK75%SS/SH2.5	100	300	125	375	150	2.5	500	1000
FA0%MK100%SS/SH2.5	0	400	125	375	150	2.5	500	1000
FA100%MK0%SS/SH2.0	400	0	125	350	175	2	500	1000
FA75%MK25%SS/SH2.0	300	100	125	350	175	2	500	1000
FA50%MK50%SS/SH2.0	200	200	125	350	175	2	500	1000
FA25%MK75%SS/SH2.0	100	300	125	350	175	2	500	1000
FA0%MK100%SS/SH2.0	0	400	125	350	175	2	500	1000

**Table 3:** Descriptive predictive performance for the experimental evaluation set comprising three newly evaluated and two independently reproduced mixture compositions.

Response	*N*	External *R*^2^	RMSE (MPa)	MAE (MPa)	MAPE (%)	NRMSE (%)	Mean Bias (MPa)	Strict PI Coverage (%)	Adjusted PI Coverage (%)
CS24	5	0.9989	0.1715	0.1323	0.47	1.38	0.0525	100	100
CS300	5	0.9062	0.2582	0.1509	1.12	10.61	0.0879	80	80
STS24	5	0.9809	0.0462	0.0411	1.40	5.04	−0.0192	100	100
STS300	5	0.9795	0.0230	0.0188	1.25	5.39	−0.0158	80	100

## Data Availability

The raw data supporting the conclusions of this article will be made available by the authors on request.
